# Klebicin E, a pore-forming bacteriocin of *Klebsiella pneumoniae*, exploits the porin OmpC and the Ton system for translocation

**DOI:** 10.1016/j.jbc.2024.105694

**Published:** 2024-01-30

**Authors:** Xinxin Zhao, Wenyu Wang, Xiaoli Zeng, Rong Xu, Bing Yuan, Wenyao Yu, Mingshu Wang, Renyong Jia, Shun Chen, Dekang Zhu, Mafeng Liu, Qiao Yang, Ying Wu, Shaqiu Zhang, Juan Huang, Xumin Ou, Di Sun, Anchun Cheng

**Affiliations:** 1 Research Center of Avian Diseases, College of Veterinary Medicine, Sichuan Agricultural University, Chengdu, Sichuan, China; 2 Key Laboratory of Animal Disease and Human Health of Sichuan Province, Chengdu, Sichuan, China; 3 Institute of Veterinary Medicine and Immunology, College of Veterinary Medicine, Sichuan Agricultural University, Chengdu, Sichuan, China; 4 Engineering Research Center of Southwest Animal Disease Prevention and Control Technology, Ministry of Education of the People's Republic of China, Chengdu, Sichuan, China; 5 Songshan Lake Materials Laboratory, Dongguan, Guangdong, China

**Keywords:** bacteriocin, klebicin E, *Klebsiella pneumoniae*, pore-forming, TonB, OmpC

## Abstract

Bacteriocins, which have narrow-spectrum activity and limited adverse effects, are promising alternatives to antibiotics. In this study, we identified klebicin E (KlebE), a small bacteriocin derived from *Klebsiella pneumoniae*. KlebE exhibited strong efficacy against multidrug-resistant *K. pneumoniae* isolates and conferred a significant growth advantage to the producing strain during intraspecies competition. A giant unilamellar vesicle leakage assay demonstrated the unique membrane permeabilization effect of KlebE, suggesting that it is a pore-forming toxin. In addition to a C-terminal toxic domain, KlebE also has a disordered N-terminal domain and a globular central domain. Pulldown assays and soft agar overlay experiments revealed the essential role of the outer membrane porin OmpC and the Ton system in KlebE recognition and cytotoxicity. Strong binding between KlebE and both OmpC and TonB was observed. The TonB-box, a crucial component of the toxin-TonB interaction, was identified as the 7-amino acid sequence (E3ETLTVV9) located in the N-terminal region. Further studies showed that a region near the bottom of the central domain of KlebE plays a primary role in recognizing OmpC, with eight residues surrounding this region identified as essential for KlebE toxicity. Finally, based on the discrepancies in OmpC sequences between the KlebE-resistant and sensitive strains, it was found that the 91st residue of OmpC, an aspartic acid residue, is a key determinant of KlebE toxicity. The identification and characterization of this toxin will facilitate the development of bacteriocin-based therapies targeting multidrug-resistant *K. pneumoniae* infections.

Microorganisms in the natural environment are important sources of novel antibacterial compounds, including antibiotics, bacteriocins, and phages (1, 2). Antibiotics have revolutionized medicine by enabling the cure of bacterial infections that were once fatal. However, the misuse of antibiotics in animal feed and the overprescription of antibiotics to humans in clinical settings have fueled the emergence and spread of antimicrobial resistance (AMR), which is posing an increasing threat to global health (3). Mortality due to AMR is estimated to reach 10 million deaths every year worldwide by 2050, surpassing the number of deaths caused by cardiovascular disorders and cancer (4). The World Health Organization recently identified the top 9 high-priority pathogens that are a major concern of humankind due to AMR. The top three pathogens are *Acinetobacter*, *Pseudomonas*, and various Enterobacteriaceae, which include *Escherichia coli*, *Klebsiella*, and *Serratia* (5). Furthermore, only a few antibiotics are currently under development in clinical trials (6). Therefore, the need to develop innovative antimicrobial therapies to address multidrug-resistant (MDR) bacterial infections is increasing.

Bacteria commonly reside in complex and diverse microbial communities where they compete for growth niches and scarce resources (7). Mechanistic studies of interbacterial competition have identified various antibacterial protein toxins, including bacteriocins, which are secreted by many species to kill competitors. Bacteriocinogenic cells synthesize a specific immune protein that acts against auto killing (7). Bacteriocins are a heterogeneous group of diffusible antimicrobial compounds that are synthesized by ribosomes. Compared to antibiotics, bacteriocins from gram-negative bacteria generally have a narrower spectrum, targeting only closely related bacteria without disrupting the microbiota, which is crucial for overall human health. As such, bacteriocins have attracted interest as platforms for novel antibacterial therapies (8, 9). For example, therapeutic administration of the probiotic bacterium *E. coli*, which produces the microcins M and H47, has been shown to limit the growth of adherent-invasive *E. coli* and *Salmonella enterica* in the inflamed gut (10).

Understanding the molecular mechanisms of toxin–bacterium interactions is essential for the application of bacteriocins in medicine and biotechnology. The bacteriocins produced by *E. coli* are called colicins (>10 kDa) or microcins (<10 kDa). Colicin-like bacteriocins from other gram-negative bacteria are named after their respective species (*e.g.*, pyocins from *Pseudomonas* and klebicins from *Klebsiella*) (11). Colicins are usually plasmid-encoded and lack signal peptides. They are generally exported from toxin-producing cells *via* release proteins (12). While colicins exhibit diverse sequences, sizes, and structures, they share a common multidomain architecture that includes an N-terminal translocation domain, a central receptor-binding domain, and a C-terminal cytotoxic domain (13). To penetrate the outer membrane barrier of target bacteria, colicins utilize cell surface receptors *via* their receptor-binding domains. These receptors are typically porins or outer membrane proteins (OMPs) involved in nutrient uptake (8). For instance, E-type colicins recognize the vitamin B12 transporter BtuB, while colicin N (ColN) binds to the porin OmpF (14). These receptors facilitate attachment, but the translocation of colicins requires the recruitment of another OMP through the translocation domain. The translocation is carried out in conjunction with either the Tol system (group A colicins) or the Ton system (group B colicins) (15, 16). Upon internalization, toxic domains exhibit three distinct cytotoxic activities: nuclease activity, pore formation, and inhibition of cell wall biosynthesis. Nuclease colicins, such as klebicin C (17) and colicins E2-E9 (18), degrade the DNA or RNA of target cells. Pore-forming colicins, including colicin E1 (ColE1), ColA, colicin-Ib, and ColN (19), disrupt the integrity of the cell membrane, leading to cell death through membrane depolarization. A few bacteriocins, such as colicin M, inhibit cell wall biosynthesis by degrading undecaprenyl phosphate-linked peptidoglycan precursors (20).

Bacteriocins are present in all major bacterial groups (7). While colicin has been extensively studied in *E. coli*, bacteriocins in other members of Enterobacteriaceae require further elucidation. In this study, we identified a protein toxin in *Klebsiella pneumoniae* through in silico analysis. The purified toxin exhibited strong antibacterial effects on MDR *K. pneumoniae* clinical isolates. Furthermore, the toxin gene conferred a growth advantage to the producer strain during interbacterial competition. These findings confirmed that the toxin is a protein bacteriocin named klebicin E (KlebE). The mode of action of KlebE and its associated membrane proteins for translocation were then systematically and thoroughly investigated.

## Results

### Screening for novel bacteriocins from *Klebsiella* and *Salmonella* strains

**Figure 1:**
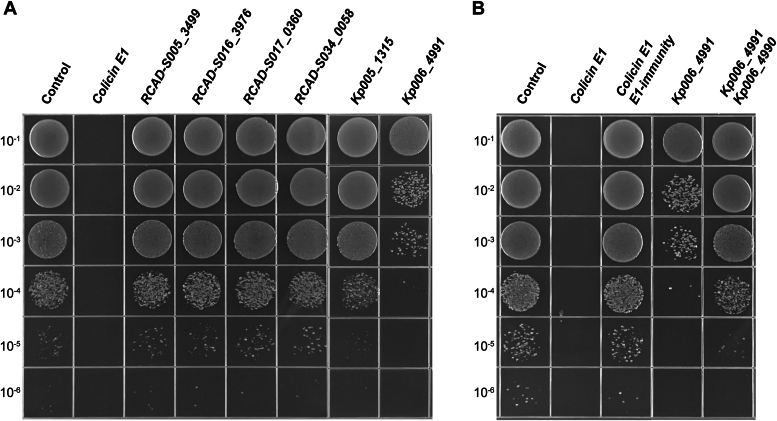
**Identification of candidate toxin and immunity genes.***A*, screening for candidate toxin genes. Recombinant *E. coli* BL21 (DE3) cells containing each of the candidate toxin genes or the positive control ColE1 gene (GenBank: WP_072647671.1) were cultured until the OD_600_ reached 0.1. Subsequently, the cultures were incubated at 37 °C with 0.1 mM IPTG for 3 h at 37 °C. Next, 10 μl of the serially diluted product was spotted onto LB agar plates and incubated overnight. Six representative candidate toxin genes are shown in this figure: *RCAD-S005_3499* (WP_001423373.1), *RCAD-S016_3976* (WP_227649104.1), *RCAD-S017_0360* (WP_241584279.1), *RCAD-S034_0058* (WP_000747895.1), *Kp005_1315* (MEC7317124.1), and *Kp006_4991* (MEC7341763.1). *B*, identification of the function of the *Kp006_4990* gene. The growth of recombinant *E. coli* BL21 (DE3) cells expressing the *Kp006_4991* gene or both the *Kp006_4991* and *Kp006_4990* genes (MEC7341762.1) was detected as described in *A*. ColE1 and its immunity gene (WP_089576385.1) were included as positive controls. Images are representative of triplicate independent experiments.

To identify novel bacteriocins with antimicrobial properties, we analyzed the genome sequences of 61 clinical *Salmonella* isolates (21) and 7 *K. pneumoniae* strains isolated from ducks in Sichuan Province, China (Table S1). In silico analyses were conducted using three antimicrobial peptide databases: BACTIBASE (22) (http://bactibase.hammamilab.org/), Bagel4 (23) (http://bagel4.molgenrug.nl/), and APD3 (24) (https://wangapd3.com/main.php/). After the removal of known bacteriocins, a total of 31 potential protein toxin sequences were obtained. These sequences were further analyzed through bactericidal assays. The pET28a plasmid harboring each candidate gene or the positive control for the ColE1 gene was transformed into the *E. coli* BL21(DE3) strain. Subsequently, bacterial growth was observed after the addition of isopropyl-β-D-1-thiogalactopyranoside (IPTG). The strain with the empty plasmid showed strong growth on Luria–Bertani (LB) agar, while no bacterial growth was observed in the ColE1 group (Fig. 1*A*). All potential toxin genes, except for *Kp006_4991,* which is located on a plasmid of the *K. pneumoniae* Kp006 strain, did not affect bacterial growth. The bacteria expressing *Kp006_4991* exhibited a significantly impaired growth phenotype (approximately 100-fold reduction) compared to that of the negative control bacteria (Fig. 1*A*), indicating that *Kp006_4991* is a toxin gene. Previous research has shown that immunity genes are generally near toxin genes (25). Therefore, we investigated the role of the downstream *Kp006_4990* gene. The introduction of *Kp006_4990* into bacteria expressing *Kp006_4991* blocked toxin-induced growth inhibition, similar to the effect of the ColE1 immunity gene (Fig. 1*B*). These findings suggested that *Kp006_4991* encodes a protein toxin, while *Kp006_4990* functions as the corresponding immunity gene.

**Figure 2:**
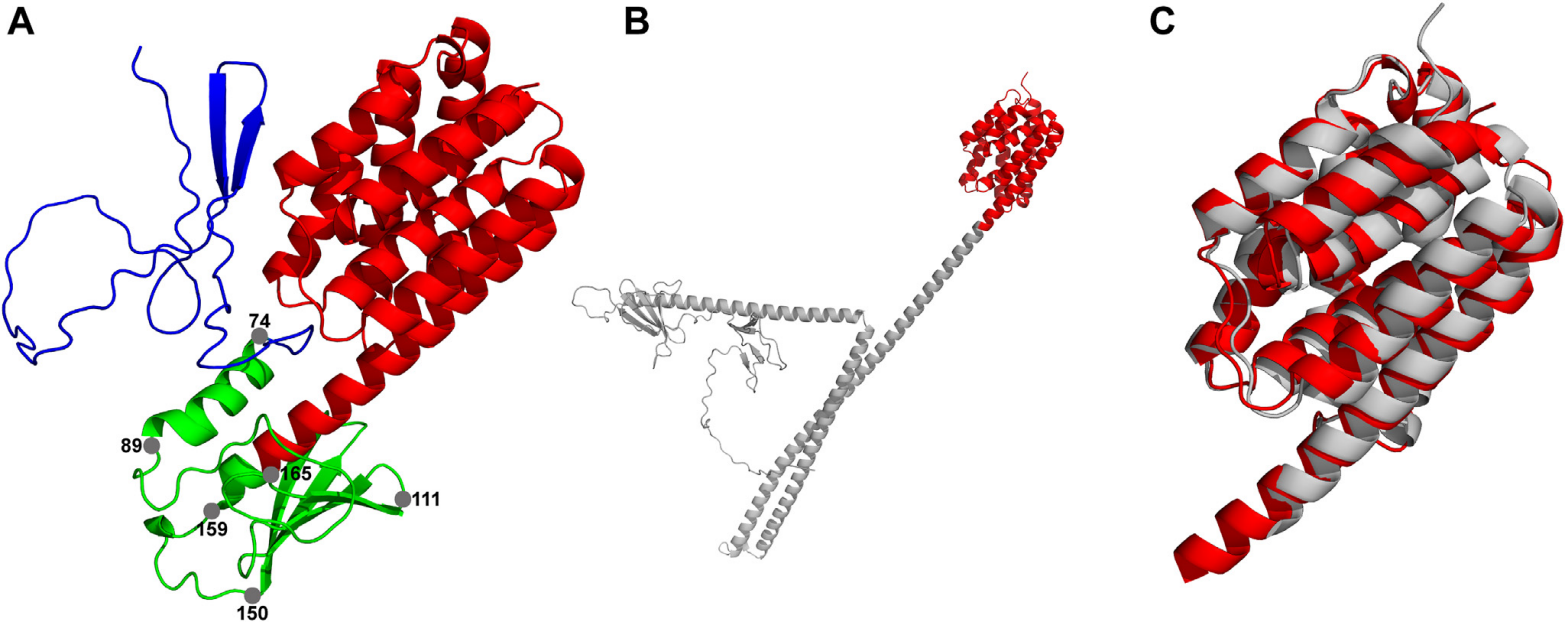
**Tertiary structures of KlebE and ColA.***A*, the tertiary structure of KlebE. The KlebE structure was predicted using AlphaFold2, and three distinct regions are visually represented with different colors. *Blue*, the disordered N-terminal domain (1–74); *green*, the globular central domain (75–165); *red*, the C-terminal toxin domain (166–377). *B*, the tertiary structure of ColA. The N-terminal translocation domain and the central receptor-binding domain (1–392) are *gray*; the C-terminal cytotoxic domain (393–595) is *red*. *C*, superimposition of the toxin domains of KlebE and ColA. *Red*, KlebE toxic domain; *gray*, ColA toxic domain.

A BLAST analysis showed that Kp006_4991 shared 41.3% identity and 51% coverage of the amino acid sequence with ColA (UniProt: P04480), 27.5% identity and 47% coverage with Col10 (UniPort: Q47125), and 25.0% identity and 41% coverage with ColΙb (UniPort: P04479) (Fig. S1). The similarity between Kp006_4991 and ColA was primarily concentrated within the C-terminal region, which includes the pore-forming domain of ColA (26). Furthermore, InterPro (27) analysis revealed only one toxic domain (pore-forming domain: IPR038283) spanning residues 159 to 373 within Kp006_4991. The crystal structure of the pore-forming fragment of ColA was previously determined (28). We aimed to predict the tertiary structure of Kp006_4991 using AlphaFold2 (29). The structure was found to consist of three distinct regions: a disordered N-terminal domain (1–74), a globular central domain (75–165) containing a 14-residue segment with high alpha-helical propensity, and a beta-sheet region spanning 40 residues flanked by two disordered regions (89–111 and 150–159). Additionally, the C-terminal domain (166–377) exhibited a pronounced alpha-helical structure (Fig. 2*A*). In contrast, the intermediate domain of ColA contains an elongated helical region with two interruptions in the helical pattern between residues 222 to 224 and 291 to 296, forming a helical hairpin structure (Fig. 2*B*). However, upon comparison of the two C-terminal domains of Kp006_4991 and ColA, we found that they share remarkably similar fundamental architectures. This architectural arrangement comprises a bundle of eight amphipathic α-helices surrounding two hydrophobic α-helices, which are completely buried within the protein (Fig. 2*C*). Thus, Kp006_4991 consists of typical tripartite domains similar to those of other colicins, and the C-terminal domain is a colicin A-like pore-forming domain. Additionally, sequence analysis and comparison revealed that the immunity protein Kp006_4990 is a tetraspan transmembrane protein with a molecular weight of 20.6 kDa that shares 17.3% and 15.9% identities with the immune protein of ColB and the immune protein of ColA, respectively (Fig. S2).

### Kp006_4991 is a bacteriocin that mediates intraspecies competition among *K. pneumoniae* species

**Figure 3:**
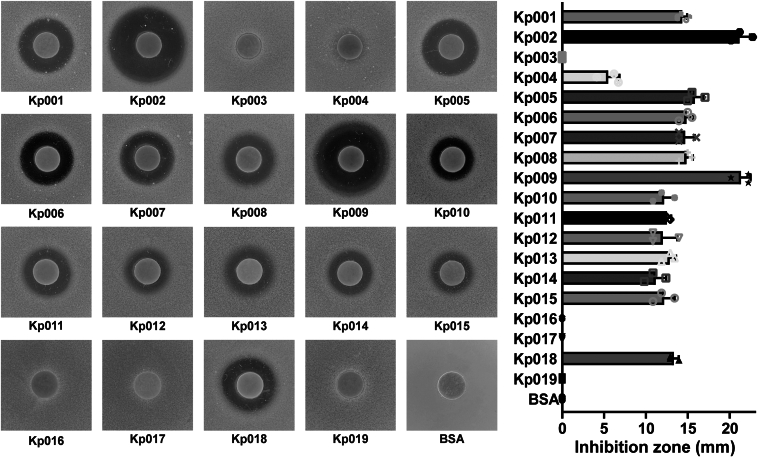
**Evaluation of KlebE activity against *K. pneumoniae* strains.** A soft agar overlay assay was used to detect the antibacterial activity of KlebE. Briefly, 20 μl of 40 μg of purified protein or BSA solution was added to various *K. pneumoniae* lawns, after which the diameters of the inhibition zones were measured following overnight incubation. The data are presented as the mean ± S.D. of triplicate independent biological replicates.

The antibiotic resistance phenotypes of 19 *K. pneumoniae* strains isolated from ducks or geese (Table S1) were determined using the minimal inhibitory concentration (MIC) assay. All 19 strains were identified as MDR strains, with eight of these strains demonstrating resistance to all 10 tested antibiotics (Fig. S3). To determine whether Kp006_4991 is a bacteriocin, we expressed and purified the toxin, which exhibited a predicted molecular weight of 39.9 kDa, from *E. coli* cells (Fig. S4). Its killing activity against these *K. pneumoniae* strains was evaluated using soft agar overlay assays. Compared to the control protein BSA, Kp006_4991 displayed activity against 15 of the 19 *K. pneumoniae* strains (Fig. 3). Varying sizes of inhibition zones were observed for the susceptible strains. The two largest zones, measuring 21.17 ± 1.26 mm and 21.33 ± 1.15 mm in diameter, were identified for strains Kp002 and Kp009, respectively. The former strain, Kp002, was selected as the indicator strain for subsequent experiments.

**Figure 4:**
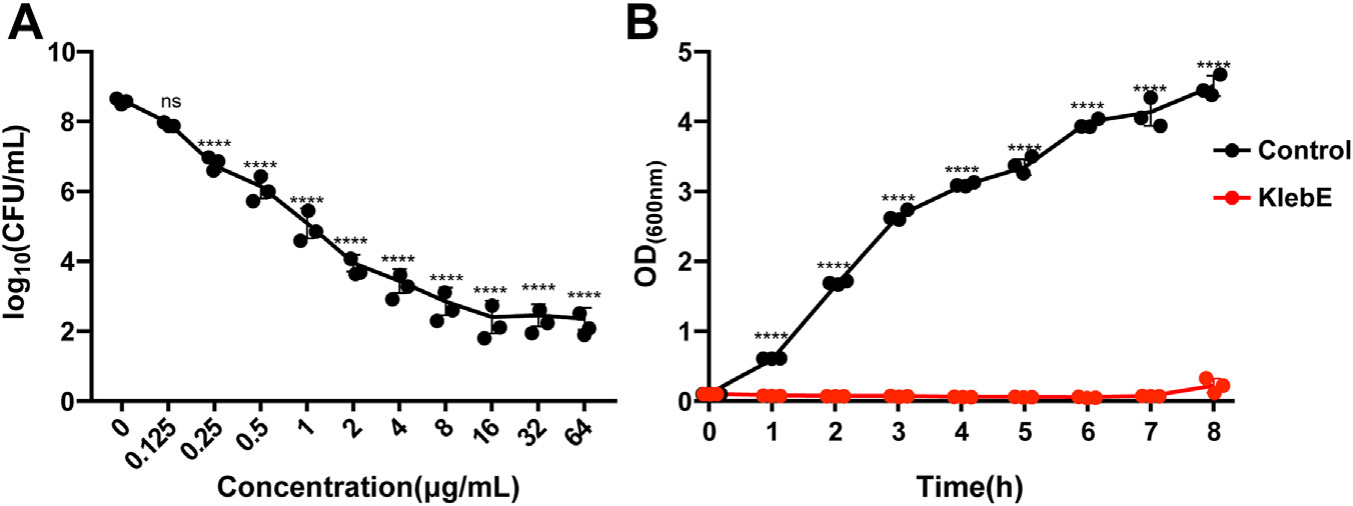
**Bactericidal properties of KlebE.***A*, KlebE dose and killing effects. The indicator Kp002 at an OD_600_ = 0.8 was incubated with different concentrations of purified KlebE ranging from 0 to 64 μg/ml at 37 °C for 1 h. The treated cells were then serially diluted 10-fold and streaked onto LB agar plates for CFU measurement. *B*, time and killing effects of KlebE. The indicator at OD_600_= 0.1 was treated with or without 16 μg/ml KlebE at 37 °C with shaking at 180 rpm. The OD_600_ was then measured every 1 h over 8 h. The data are shown as the mean ± S.D. of three independent biological replicates and were analyzed by one-way ANOVA followed by Dunnett's multiple comparison test (*A*) or two-tailed Student’s *t* test (*B*). The *asterisk* above the error bar indicates a significant difference compared with the control group. ns, not significant; ∗∗∗∗*p* < 0.0001.

To assess the bactericidal properties, we incubated approximately 10^8^ CFU of the indicator with progressively increasing concentrations of KlebE for 1 h at 37 °C, after which the bacterial CFUs were quantified. This indicator exhibited dose-dependent susceptibility to KlebE (Fig. 4*A*). A concentration equal to or exceeding 16 μg/ml demonstrated the most pronounced bactericidal effect, resulting in a reduction in cell count of approximately 6.20 log units. Additionally, KlebE was effective at relatively lower concentrations ranging from 0.25 to 1.0 μg/ml. Notably, treatment with 1 μg/ml KlebE eradicated approximately 3.52 log units of the indicator strain (Fig. 4*A*). Furthermore, lethal effects were observed during cellular growth after exposure to 16 μg/ml KlebE toxin. In the absence of KlebE, the bacteria exhibited typical growth patterns, with the OD_600_ increasing from 0.1 to 4.51 ± 0.15; however, exposure to the toxin resulted in no growth throughout the entire observation period of 8 hours (Fig. 4*B*). Consequently, KlebE had a strong bactericidal effect on susceptible strains of *K. pneumoniae*.

**Figure 5:**
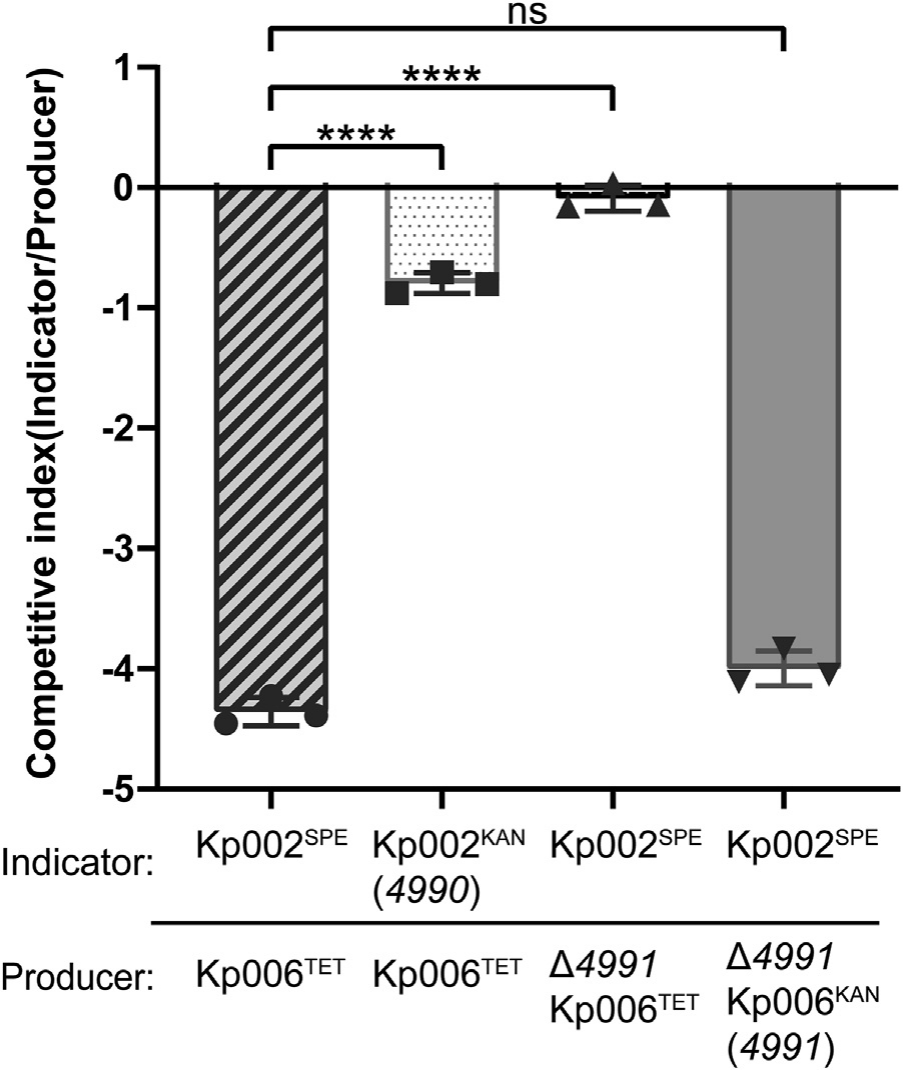
**Interbacterial competition of *K. pneumoniae*.** The producer and indicator strains depicted in this figure were mixed in a 1:1 ratio and cultivated for 12 h in LB medium at 37 °C. The culture medium was subsequently diluted and applied to LB agar plates supplemented with appropriate antibiotics to quantify the population of each strain. The specific antibiotics used for each strain are indicated by superscripts. The CI was calculated as the final frequency of the indicator divided by its initial frequency in the mixed cocultures. The data are presented as the mean ± S.D. of three independent biological replicates and were analyzed using one-way ANOVA followed by Dunnett's multiple comparison test. An asterisk above the line indicates a significant difference between the two indicated groups. ns, not significant; ∗∗∗∗*p* < 0.0001. KAN, kanamycin; SPE, spectinomycin; TET, tetracycline.

In addition, the role of the Kp006_4991-Kp006_4990 toxin-immunity pair in interbacterial competition was determined through coculture competition assays. The producer strain Kp006 (resistant to tetracycline) was cocultured with the susceptible indicator strain Kp002 (resistant to streptomycin) for 12 h, after which the competitive index (CI) was calculated. The producer exhibited a significant growth advantage (>10^4^-fold) compared to the indicator (Fig. 5). This growth advantage was abolished upon deletion of the *Kp006_4991* gene from the producer or upon expression of the *Kp006_4990* gene in the indicator strain (Fig. 5). Complementation of *Kp006_4991* in the toxin mutant strain reinstated the growth advantage (Fig. 5). These findings suggest that the Kp006_4991 toxin enhances the growth fitness of the toxin-producing strain Kp006 by killing neighboring *K. pneumoniae* strains. Because the toxin kills bacteria in a cell contact-independent manner and because most known colicins or pyocins are >50 kDa (30), we concluded that Kp006_4991 is a small protein bacteriocin of *K*. *pneumoniae* and designated it KlebE.

### KlebE kills susceptible bacteria by disrupting the cell envelope

**Figure 6:**
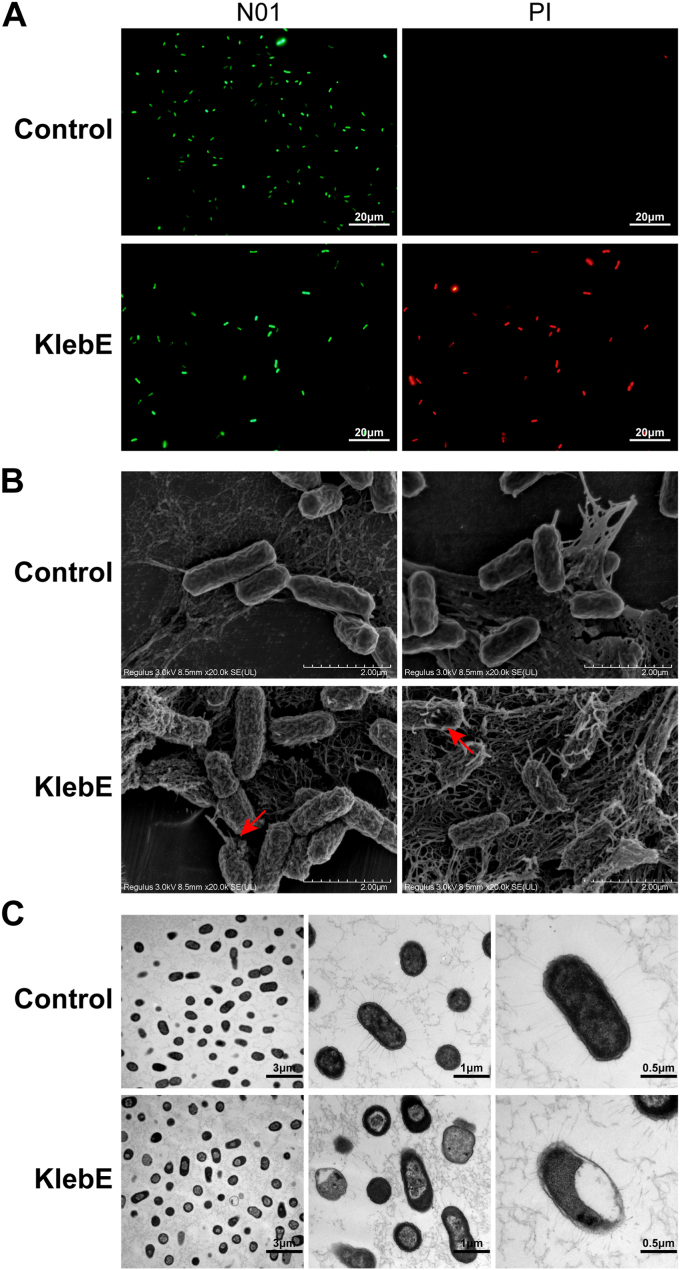
**Microscopic observations.** The indicator Kp002 in the logarithmic growth phase was incubated with 16 μg/ml KlebE or PBS for 1 h at 37 °C. Then, the bacterial cells were subjected to double staining with N01 and PI followed by fluorescence microscopy observation (*A*), SEM observation at a magnification of × 50,000 (*B*), and TEM observation (*C*). The scale bars in *A* and *B* are 20 μm and 2 μm, respectively. The scale bars in *C* from *left to right* are 3 μm, 1 μm, and 0.5 μm. The *red arrows* indicate the formed pores.

To comprehensively investigate the cytotoxic activity of KlebE, we measured the membrane permeability and cell morphology of the indicator strain following exposure to 16 μg/ml KlebE for 1 h. We conducted double staining assays using N01 (live cell probe) and PI (dead cell probe). We found that the indicator strain without toxin treatment exhibited only bright green fluorescence. However, the cells incubated with KlebE emitted both bright green and red fluorescence (Fig. 6*A*), indicating that the addition of KlebE increased the membrane permeability of the indicator. Scanning electron microscopy (SEM) and transmission electron microscopy (TEM) further revealed that KlebE significantly disrupted the integrity of the indicator cell membrane (Fig. 6, *B* and *C*). In contrast to the intact cell structures observed in the untreated bacteria, which exhibited plump rhabditiform and multilayered cell structures, the treated cells exhibited significant shrinkage of the cell membrane (Fig. 6*B*). Additionally, conspicuous surface pores were formed (Fig. 6*B*), accompanied by leakage of the cytoplasmic contents (Fig. 6, *B* and *C*). The treated cells also had thin and deformed cell membranes as well as aberrant cellular structures with loosely arranged and cavitated cytoplasm (Fig. 6*C*). Therefore, KlebE treatment has a significant adverse effect on the structural integrity of bacterial cell surfaces, as shown by the formation of pores in the cell membrane.

**Figure 7:**
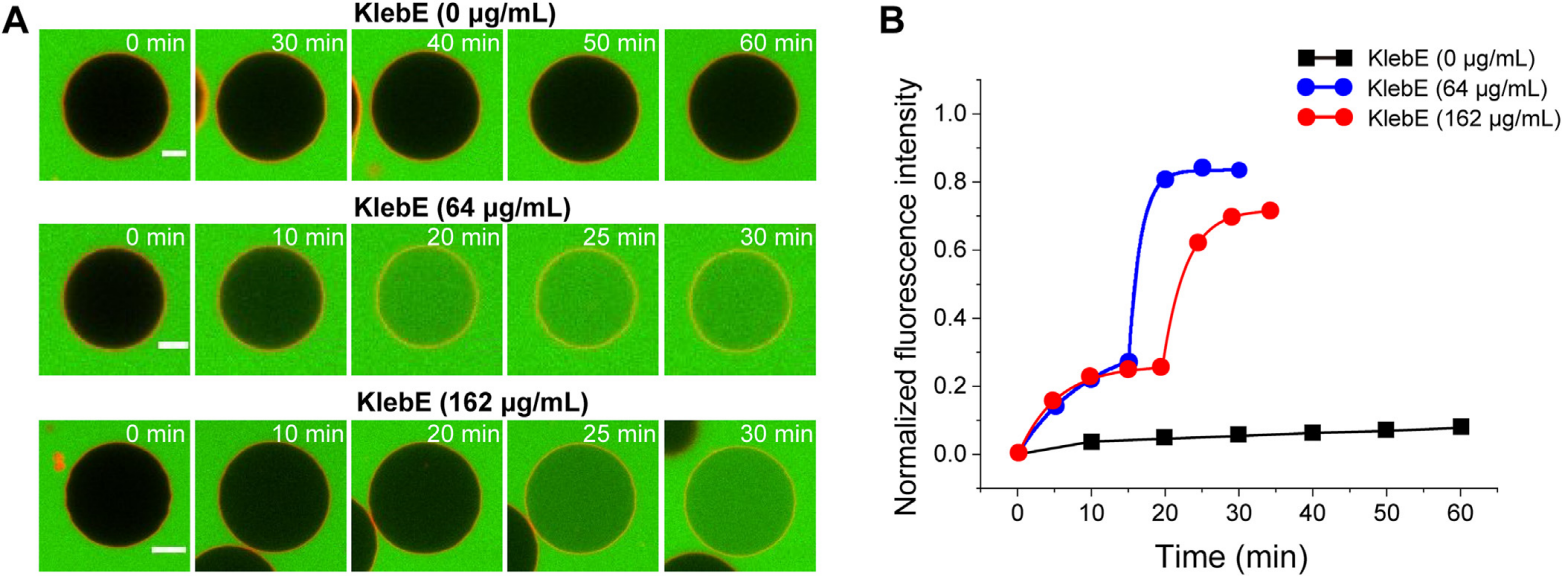
**KlebE induces GUV membrane permeabilization.***A*, Two representative GUVs demonstrating the transmembrane entry of calcein into GUVs due to exposure to 64 and 162 μg/ml KlebE. The condition without the addition of KlebE was also included as a reference. The time points after KlebE addition are marked in each image, and the scale bars indicate a length of 5 μm. *B*, corresponding time evolution of the fluorescence intensity in the interior of the GUV upon exposure to varying concentrations of KlebE. The monitoring points are fitted in stages using the sigmoidal equation of $y=\frac{a}{1+\exp\left(-k\times \left(x-x_{c}\right)\right)}$, where *a*, *k*, and *x*_*c*_ are constants, *x* is the time and y is the normalized fluorescence intensity.

The dynamic giant unilamellar vesicle (GUV) leakage assay was further employed to evaluate the interaction between KlebE and a model bacterial membrane composed of a mixture of phosphatidylcholine (PC) and phosphatidylglycerol (PG) lipids (PC/PG = 4/1 by mol) labeled with 0.1 mol% lissamine rhodamine B-phosphoethanolamine (RhB-PE). The GUVs were dispersed in calcein (a small dye molecule) solution and remained stable without calcein entry for 4 h (Fig. 7*A*). However, the addition of KlebE induced transmembrane entry of calcein into the interior of GUVs. Specifically, after exposure to 64 μg/ml KlebE, one-third of the GUVs (in three independent experiments) exhibited permeation along with entry of calcein. In contrast, exposure to a high concentration of 162 μg/ml KlebE resulted in calcein entry into all the observed GUVs across three independent experiments. To quantitatively assess the efficiency of KlebE in permeabilizing membranes, we acquired time-dependent fluorescence intensity profiles within individual vesicles, which are shown in Figure 7*B*. Intriguingly, the fluorescence intensity gradually increased, followed by an abrupt increase until it reached saturation. This process significantly differs from that observed with conventional antimicrobial peptides (*e.g.*, melittin, magainin, PGLa, or LL-37) or nanoparticles (*e.g.*, fullerene or graphene oxide nanosheets) (31, 32), which exhibit an obvious sigmoidal distribution curve. The observed process can be accurately described by two sigmoidal equations before and after the transition time point (*e.g.*, at 20–25 min at 162 μg/ml). Moreover, there was no discernible variation at this critical time point across the different protein concentrations (*i.e.*, 64 and 162 μg/ml). In summary, GUV leakage assays have demonstrated that KlebE is a pore-forming toxin that has unique membrane permeabilization effects and has a mechanism of action distinct from that of typical antimicrobial peptides or materials.

### The OmpC and Ton systems are required for KlebE toxicity

**Figure 8:**
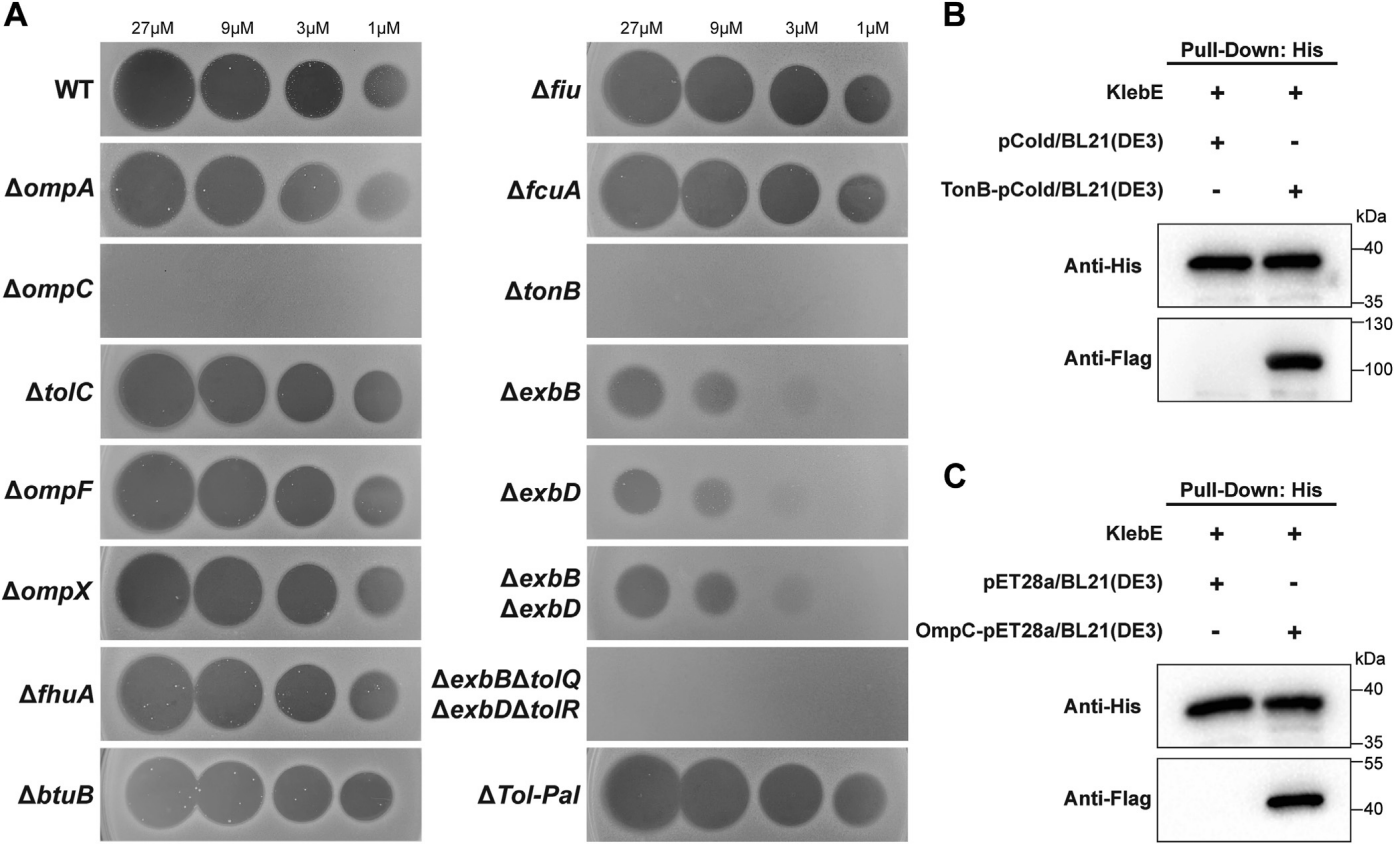
**Identification of the membrane proteins required for KlebE toxicity.***A*, Soft-agar overlay assay. The cytotoxicity of KlebE was evaluated by spotting it onto soft-agar lawns inoculated with the indicator strain Kp002 or various mutant strains derived from Kp002 at concentrations of 27, 9, 3, and 1 μM. *B* and *C*, detection of protein interactions. The interactions between KlebE_-His_ and whole-cell lysates of BL21 (DE3) harboring the plasmid pCold or TonB-pCold (*B*), as well as *E. coli* cells with or without OmpC_-Flag_ expression (*C*), were assessed using pulldown assays and subsequent Western blotting with a His_6_-specific mAb and a Flag-specific mAb.

To elucidate the import mechanism of KlebE, we employed pulldown assays to identify its outer membrane (OM) receptor or translocator. Specifically, purified KlebE was incubated with lysed whole-cell proteins of Kp002 and subjected to protein A/G affinity chromatography, sodium dodecyl sulfate‒polyacrylamide gel electrophoresis (SDS‒PAGE), and peptide mass fingerprinting. This approach led to the identification of four membrane proteins: OmpA, OmpC, TolC, and TolB. Subsequently, we evaluated the lethal effects of KlebE on *ompA*, *ompC*, and *tolC* mutant strains using overlay assays. At concentrations of 27 μM, 9 μM, 3 μM, and 1 μM, KlebE produced inhibition zones of comparable sizes on the Δ*ompA*, Δ*tolC*, and WT strains. However, complete resistance to KlebE was observed in the Δ*ompC* strain (Fig. 8A). Furthermore, we investigated the roles of other OM receptors/translocators (18) of colicins that had been previously identified. Our results demonstrated that deletion of *ompF*, *ompX*, *fhuA*, *btuB*, *fiu*, or *fcuA* did not affect the toxicity of KlebE (Fig. 8*A*). These findings strongly suggested that OmpC functions as a receptor or translocator for KlebE import. Given that colicins utilize either the Tol system or Ton system for translocation, we knocked out genes involved in the Tol system (*tolA*, *tolB*, *tolQ*, *tolR*, and *pal*) and Ton system (*tonB*, *exbB*, and *exbD*) in the indicator strain. We observed that the inhibition zones in the Δ*exbB* and Δ*exbD* strains were significantly smaller than those in the WT strain and vanished entirely in the Δ*tonB* strain. However, the Δ*Tol-pal* strain remained susceptible (Fig. 8*A*). Interestingly, the duple mutant (Δ*exbB*Δ*exbD*) was still sensitive to KlebE, similar to Δ*exbB* or Δ*exbD*, but it became fully resistant with further deletions of *tolQ* and *tolR* (Fig. 8*A*). Therefore, we concluded that KlebE is a group B bacteriocin dependent on the OmpC and the Ton system. Further evidence supporting this conclusion was provided by pulldown assays, which demonstrated the strong binding of purified KlebE_-His_ to both the TonB_-Flag_ and OmpC_-Flag_ proteins (Fig. 8, *B* and *C*).

### A TonB box is present in the disordered N-terminus of KlebE

**Figure 9:**
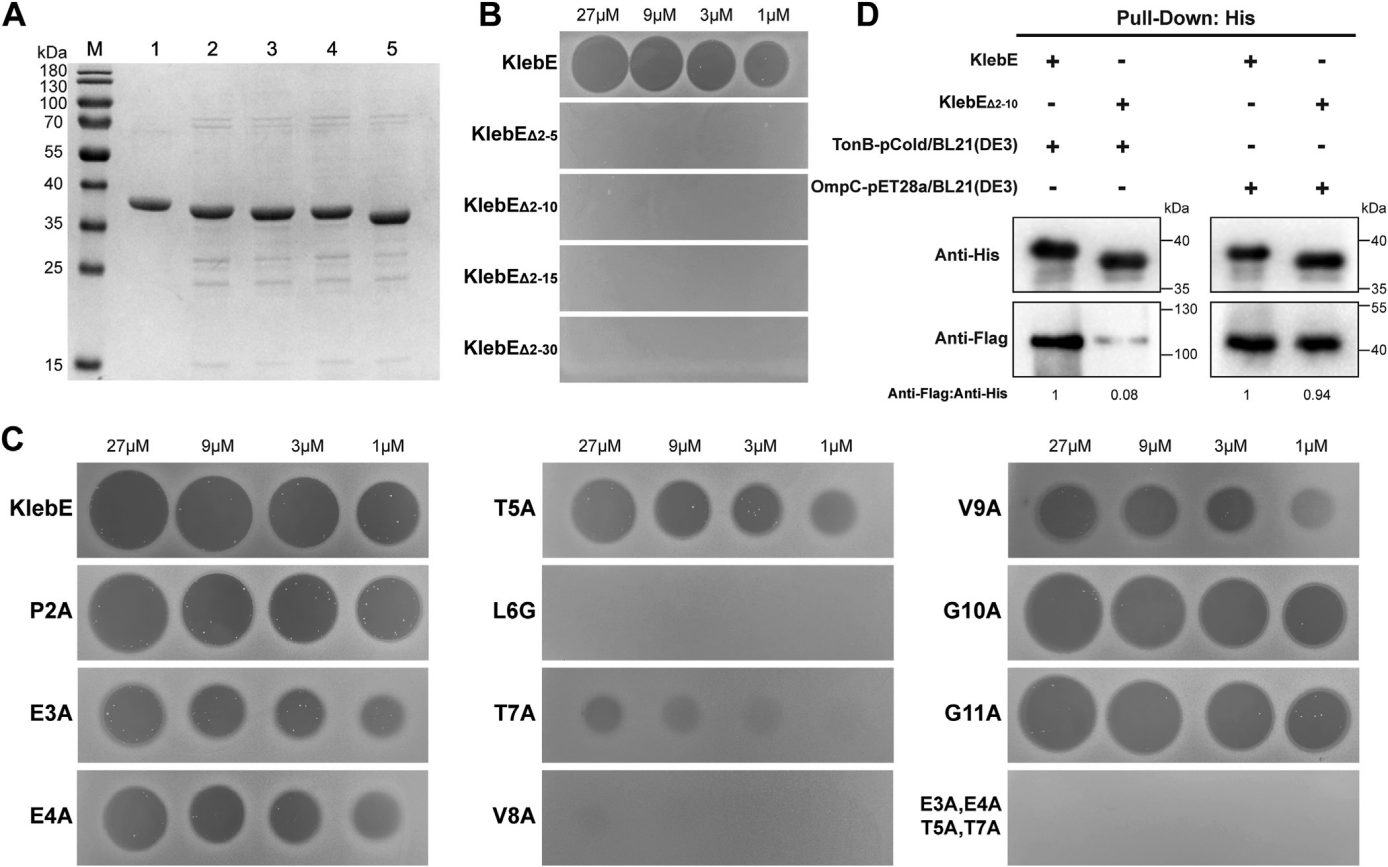
**Determination of the location of the TonB-box of KlebE.***A*, SDS‒PAGE analysis of purified truncated KlebE proteins. Lanes: M, protein marker; 1, KlebE; 2, KlebE_Δ2-5_; 3, KlebE_Δ2-10_; 4, KlebE_Δ2-15_; 5, KlebE_Δ2-30_. *B* and *C*, Soft-agar overlay assay. The cytotoxicities of complete KlebE, truncated KlebE (*B*) and the mutant KlebE (*C*) were assessed by spotting them onto soft agar lawns inoculated with the indicator Kp002 at concentrations of 27, 9, 3, and 1 μM. *D*, detection of protein interactions. The interactions between complete KlebE_-His_ or KlebE_Δ2-10-His_ and TonB_-Flag_ and OmpC_-Flag_ were determined using pulldown assays and Western blotting with a His_6_ mAb and a Flag mAb. The KlebE bands specific to the anti-His_6_ mAb demonstrated similar sample loads among the groups. Densitometry analysis of protein interactions was conducted *via* Image J software.

Bacteriocins that utilize the Ton motor complex contain a recognition sequence at the N-terminus, designated the TonB-box (33). To identify the TonB-binding region of KlebE, we expressed and purified four truncated proteins with His_6_-tags: KlebE_Δ2-5_, KlebE_Δ2-10_, KlebE_Δ2-15_, and KlebE_Δ2-30_ (Fig. 9*A*). Then, we evaluated the ability of these compounds to inhibit Kp002 using overlay assays. The results indicated that none of the truncated KlebE proteins produced inhibition zones on the indicator agars (Fig. 9*B*), suggesting that the TonB box is located within the first 2 to 30 residues. Given that a typical TonB box consists of 5 to 7 amino acids, we hypothesized that this region resides within residues 2 to 10 of KlebE. To test this hypothesis, we constructed 10 point mutations in the KlebE protein spanning from the second residue (proline) to the 11th residue (glycine). We found that the P2A, G10A, and G11A mutations did not affect the toxic activity of KlebE. However, the mutations E3A, E4A, T5A, T7A, and V9A resulted in notable reductions in the sizes of the inhibition zones. Moreover, the L6G and V8A mutations completely abolished the toxicity of KlebE (Fig. 9*C*). Concurrent mutations at positions 3, 4, 5, and 7 also resulted in a loss of the bactericidal ability of KlebE (Fig. 9*C*). Therefore, the region encompassing the third to ninth residue (E^3^ETLTVV^9^) is crucial for the function of KlebE. A pulldown assay further confirmed that KlebE_Δ2-10_, which contains a His_6_-tag, could bind to the OmpC_-Flag_ protein as effectively as the wild-type KlebE but exhibited negligible binding affinity toward the TonB_-Flag_ protein (Fig. 9*D*). Thus, E^3^ETLTVV^9^ constitutes the TonB-box of KlebE, with L6 and V8 being identified as the most critical amino acids.

### Location and structure of the OmpC-binding domain of KlebE

**Figure 10:**
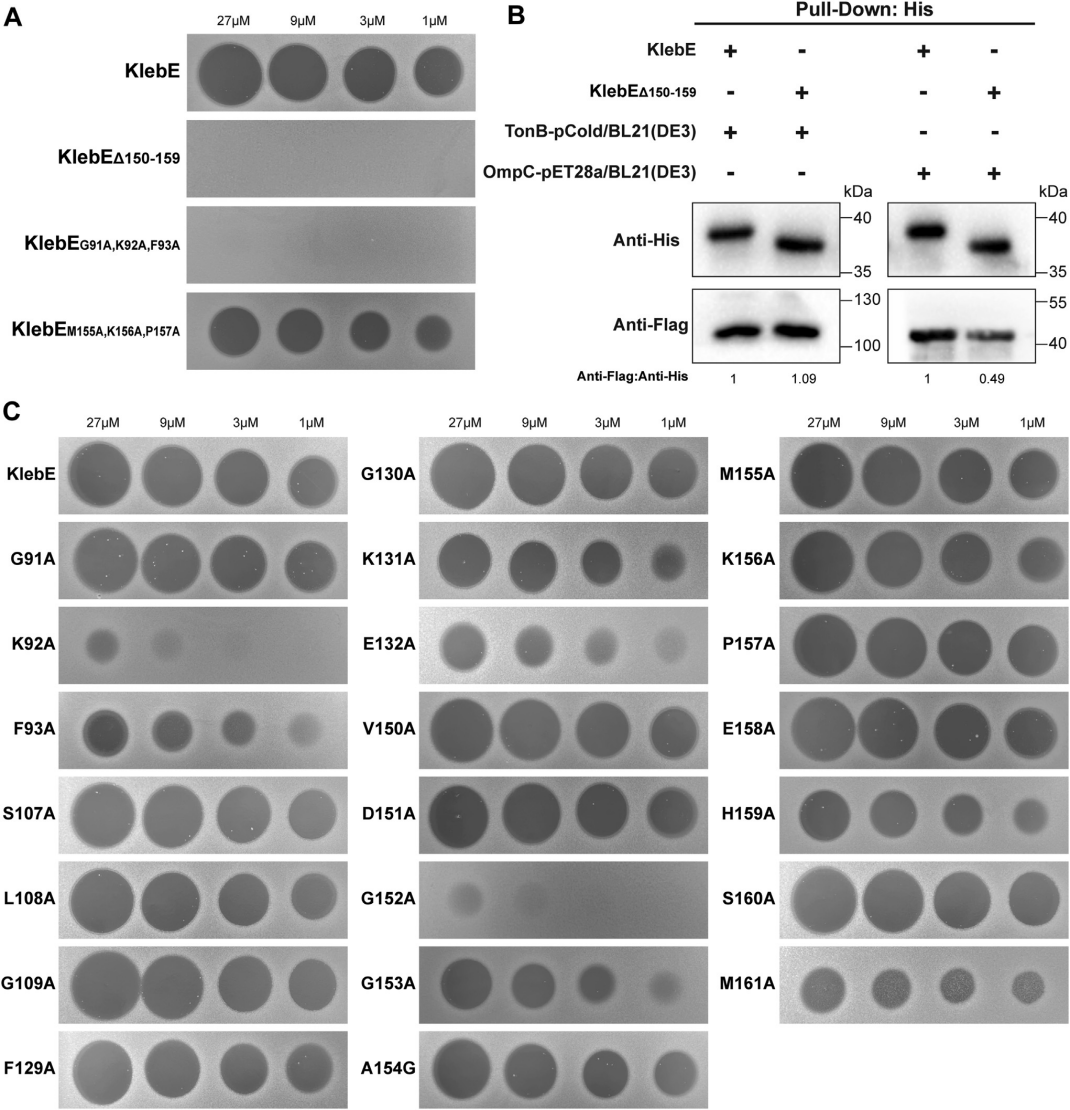
**Determination of the location of the OmpC-binding domain of KlebE.***A*, the antimicrobial effects of wild-type KlebE, KlebE_Δ150-159_, or two triple-mutant KlebEs on the indicator Kp002 were assessed using a soft agar overlay assay. *B*, the interactions of complete KlebE_-His_ or KlebE_Δ150-159-His_ with TonB_-Flag_ and OmpC_-Flag_ were determined using pulldown assays and Western blotting with a His_6_ mAb and a Flag mAb. The KlebE bands specific to the anti-His_6_ mAb demonstrated similar sample loads among the groups. Densitometry analysis of protein interactions was conducted *via* Image J software. *C*, a soft agar overlay assay was applied to detect the cytotoxicity of KlebE or KlebE with point mutations to the indicator Kp002.

The receptor binding domains of colicins are known to associate with OM receptors (34, 35). In our study, we hypothesized that the central domain (residues 75–165) of KlebE is involved in OmpC binding. To address this issue, we aimed to design and purify five truncated proteins, namely, KlebE_Δ75-165_, KlebE_Δ75-98_, KlebE_Δ88-112_, KlebE_Δ99-159_, and KlebE_Δ150-159_. However, only KlebE_Δ150-159_ was successfully purified in sufficient quantity for the overlay assay. Given that the binding site is typically located on the protein surface (36, 37), we simultaneously engineered two three-point mutants targeting the bottommost amino acid sites (A92, A156) of KlebE, namely, KlebE_G91A,K92A,F93A,_ and KlebE_M155A,K156A,P157A_. Neither KlebE_Δ150-159_ nor KlebE_G91A,K92A,F93A_ had antibacterial activity against the indicator strain, while the inhibition zone sizes of KlebE_M155A,K156A,P157A_ were slightly decreased (Fig. 10*A*). Moreover, while KlebE_Δ150-159_ demonstrated the anticipated binding affinity for TonB_-Flag_, it recognized the OmpC_-Flag_ protein less effectively than did wild-type KlebE (Fig. 10*B*). These observations indicated that residues 150 to 159 participate in the recognition of OmpC by KlebE.

**Figure 11:**
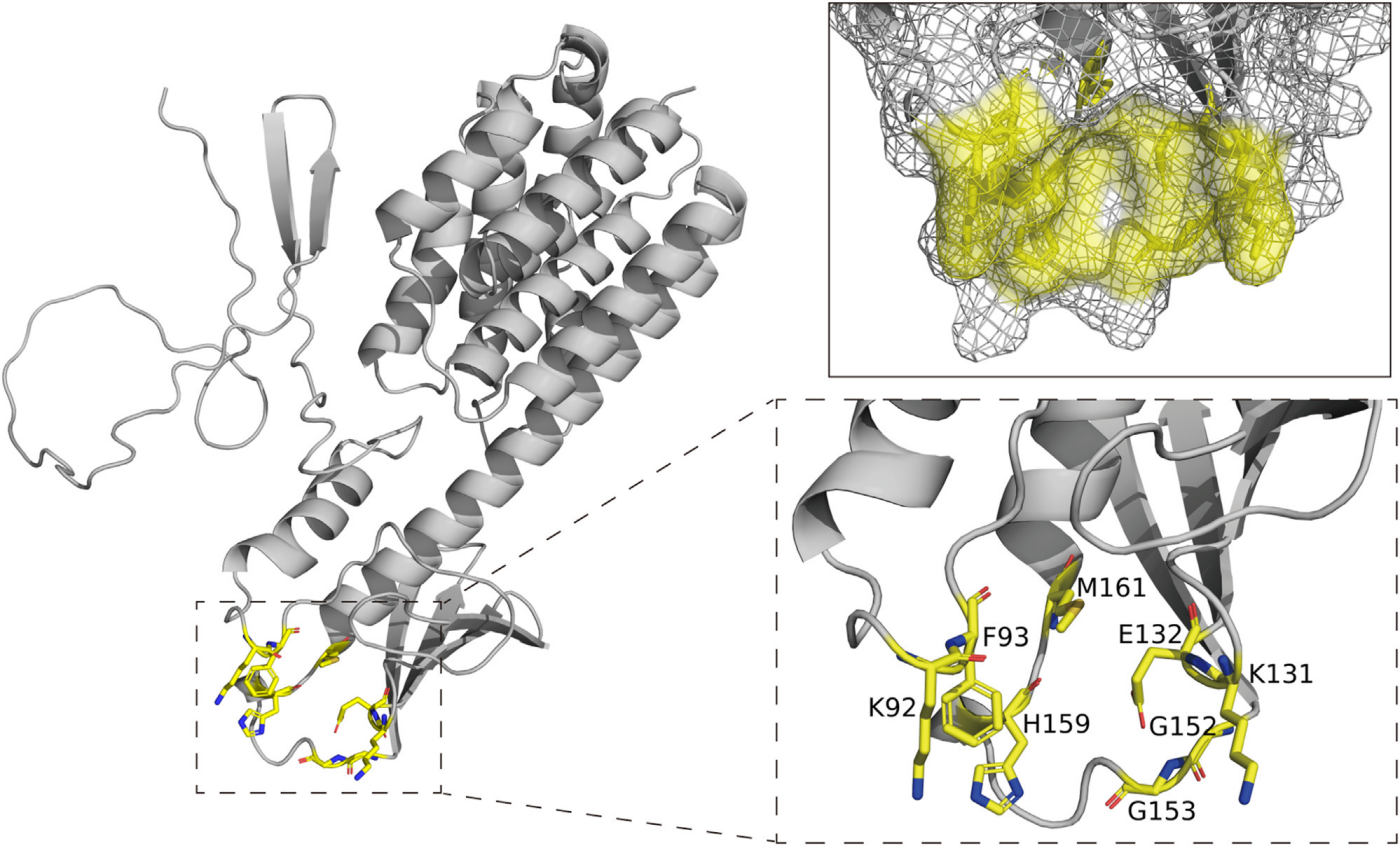
**The protein-binding pocket sites on the spatial structure of KlebE.** The amino acid residues K92, F93, K131, E132, G152, G153, H159, and M161 of KlebE are displayed using a *stick model* (*yellow*), while the remaining structures are shown using a cartoon model (*gray*) in PyMOL. The image in the *top right corner* shows the surface of the protein-binding pocket.

The ProteinsPlus tool (38, 39) was used to predict potential binding pockets of KlebE, resulting in the identification of 17 binding pockets. Among these, one specific pocket, P_10 (Fig. S5), contained essential amino acids within regions 91 to 93 and 150 to 159, indicating its potential association with OmpC binding. Further analyses of 22 point mutations around the P_10 pocket revealed that eight amino acids, K92, F93, K131, E132, G152, G153, H159, and M161, played critical roles in the toxicity of KlebE, as their corresponding mutant proteins exhibited significantly reduced inhibitory effects (Fig. 10*C*). The 92nd (lysine) and 152nd (glycine) residues were identified as the most crucial, as their point mutant proteins exhibited a nearly complete loss of antimicrobial activity. Mapping these eight residues onto the spatial structure of KlebE revealed that six residues (K92, F93, K131, E132, G152, and G153), including the most critical ones, were situated within two disordered regions in the central domain (Fig. 11). This observation underscores the importance of these regions in OmpC binding.

### Identification of key residues of OmpC involved in KlebE toxicity

**Figure 12:**
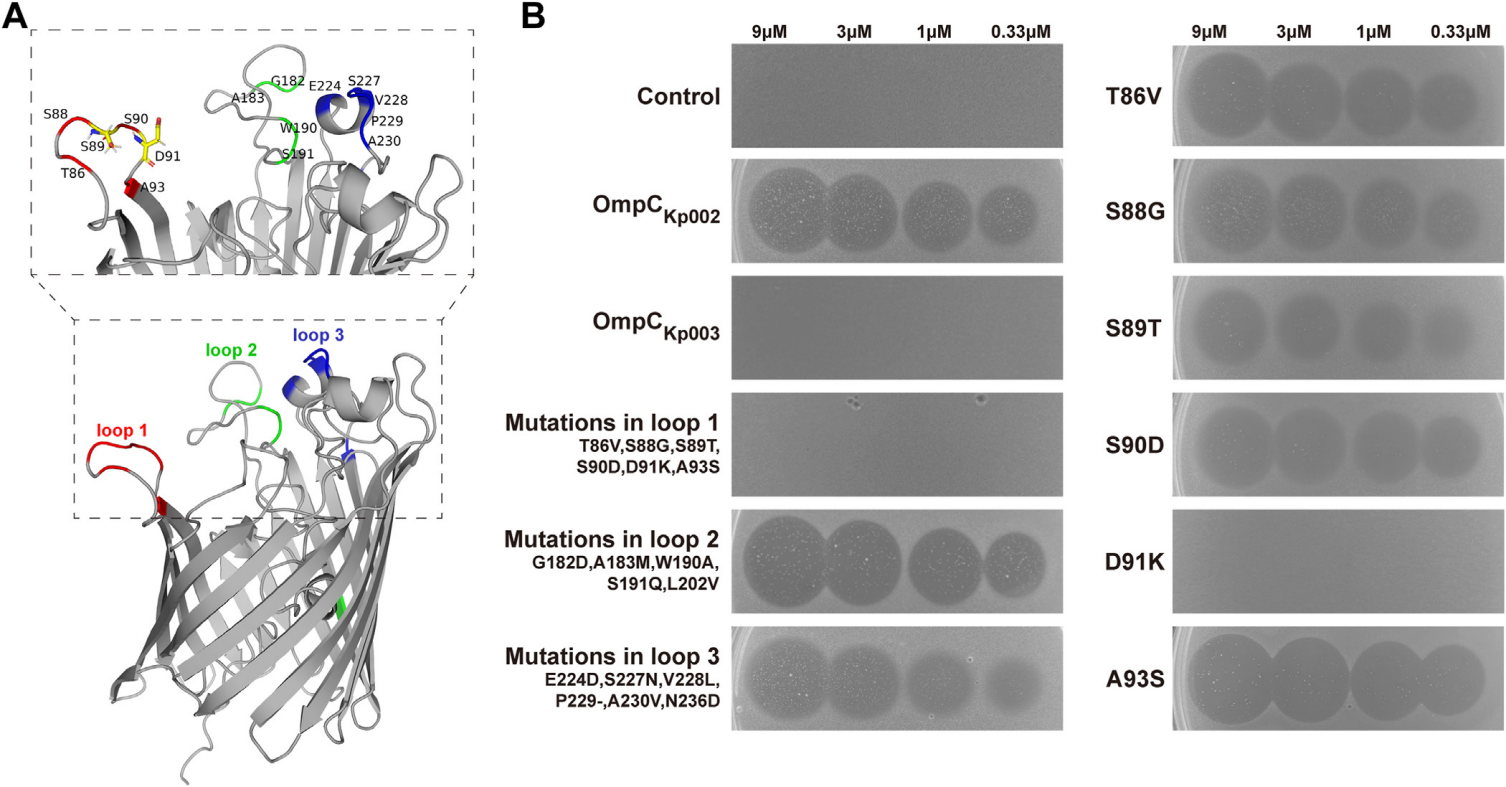
**Identification of essential residues of OmpC for KlebE toxicity.***A*, mutation sites in the predicted *K. pneumoniae* OmpC tertiary structure. The OmpC_Kp002_ structure was predicted by AlphaFold2. The mutated amino acids in the insusceptible strains are marked with colors and position numbers. *B*, soft agar overlay assay. Susceptibility to KlebE at concentrations of 9, 3, 1, and 0.33 μM was assessed for the wild-type *E. coli* BL21 (DE3) (control) or recombinant *E. coli* strains expressing OmpC_Kp003_, OmpC_Kp002_ or its derivatives with the indicated mutations, respectively.

Receptor sequences are key determinants of the host specificity of bacteriocins. BLAST analysis of the four insusceptible *K*. *pneumoniae* strains (Fig. 3, Kp003, Kp016, Kp017 and Kp019) revealed identical OmpC sequences, which exhibited 17 point mutations, in contrast to that of the indicator Kp002 (Fig. S6). These mutations were mainly distributed in or near three extracellular loops of OmpC of Kp002 (OmpC_Kp002_), named loop 1, loop 2, and loop 3; only one mutation site (L202) was present in the β sheet lumen (Fig. 12*A*). To assess the roles of these mutations in KlebE toxicity, we constructed a series of engineered *E. coli* BL21 (DE3) cells expressing different OmpC sequences. The wild-type *E. coli* strain was initially resistant to KlebE treatment, while the expression of OmpC_Kp002,_ rather than OmpC_Kp003,_ in *E. coli* conferred acquired susceptibility to the toxin (Fig. 12*B*), indicating the importance of these mutations in Kp003. Respective introduction of three multipoint mutations into OmpC_Kp002_ revealed that the *E. coli* strain with the mutations in loop 1 (T86V, S88G, S89T, S90D, D91K, A93S) became fully resistant to KlebE-mediated killing, while the other two combination mutations in loop 2, loop 3 and the lumen did not change the bacterial phenotypes to the toxin (Fig. 12*B*). Further construction and analysis of all 6 point mutations within loop 1 revealed that the S89T mutation in OmpC_Kp002_ led to mildly decreased bacterial sensitivity to the toxin, while the D91K mutation resulted in a fully resistant phenotype in *E. coli*. In contrast, the remaining 4 mutations had no effect on KlebE susceptibility (Fig. 12*B*). Therefore, the 91st amino acid, aspartic acid, of OmpC is an essential residue for KlebE toxicity.

## Discussion

*K. pneumoniae* is one of the six ESKAPE pathogens (*Enterococcus. faecium*, *Staphylococcus aureus*, *K*. *pneumoniae*, *Acinetobacter baumannii*, *Pseudomonas aeruginosa*, and *Enterobacter* spp.) that are responsible for global hospital-acquired infections and challenging to treat with antibiotic therapy (40, 41). This bacterium poses a major public health threat due to the emergence of strains resistant to most or all available antibiotics and its ability to amplify and disseminate clinically important AMR genes (42). Our study confirmed the severity of AMR in *K. pneumoniae* isolates from ducks or geese, including eight strains exhibiting resistance to all 10 tested antibiotics.

The global AMR crisis highlights the pressing need for the discovery of alternative antimicrobial agents. *Escherichia* spp. have revealed an extraordinary array of colicins, while only a limited number of bacteriocins (*e.g.*, klebicin B, klebicin C, and klebicin D) (43, 44) from *Klebsiella* species have been identified thus far. Here, we identified a protein bacteriocin called KlebE from 31 potential toxin genes. The low hit rate was likely due to the high sequence diversity of colicin or colicin-like bacteriocins resulting from mutations by evolutionary recombination and selection pressure (13). Another possibility was that some toxin genes were not expressed efficiently in the *E. coli* strain. The ability of KlebE to kill MDR *K. pneumoniae* strains and achieve a killing effect at nanomolar concentrations highlights its potential application value. Recently, the identified plant-expressed toxin KpneA (45) also demonstrated a similar antimicrobial effect against AMR *K. pneumoniae*; however, the role of KpneA in bacterial competition was not determined. Participation in bacterial competition is a property of bacteriocins. Our study employed distinct methodologies to characterize KlebE and offered a more comprehensive and thorough examination of this bacteriocin. The growth fitness provided by KlebE in interbacterial competition *in vitro* suggested that KlebE might play an important role in the ecological adaptation of *K. pneumoniae*. Further analysis of the function of KlebE in natural bacterial infection niches is important.

Immunity proteins for colicins are expressed constitutively to avoid autointoxication *via* interaction with the active sites of toxins (8). Nuclease colicins are released in complex with cognate immunity proteins that are detached before the import of toxins into target bacteria, while immunity proteins for pore-forming toxins are usually small and localize to the cytoplasmic membrane, enabling specific intramembrane helix-helix interactions with the toxins (46). The immunity protein Cai binds directly to the pore-forming domain of ColA (46). According to the number of transmembrane regions, immunity proteins of pore-forming colicins are classified into two subgroups (13): the A type (immunity proteins against colicins A, B, and N), with four transmembrane helices; and the E1 type (those against colicins E1, Ia, K, and pyocin S5), with three transmembrane helices; therefore, the immunity protein Kp006_4990 belongs to the A type.

Like well-studied colicins or pyocins, KlebE has a tripartite architecture comprising a disordered N-terminus, a central globular domain, and a C-terminal toxic domain. The evolution of colicins and the generation of numerous chimeric colicins are driven by horizontal gene transfer and the recombination of heterologous bacteriocins (33, 44, 47). Previous studies have shown that klebicin C consists of a ColE1-like translocation domain, a ColD-like receptor-binding domain, and a ColE4 killing domain (44). We observed that KlebE possesses a semiconserved C-terminal domain along with two unique domains. This finding indicates that there are potentially more bacteriocins that remain to be discovered and examined. The pore-forming domain in KlebE was predicted to have a 10-helix bundle structure, which is parallel to that in ColA. This structure is consistent with its bactericidal activity, which disrupts the cell envelope. Interestingly, similar structures were also observed for ColB, ColD, and pyocin S5 (48).

A disordered N-terminus is a characteristic structural feature of colicins (17, 48) and contains epitopes designed to interact with the energized TonB or Tol proteins in the periplasm. These proteins play crucial physiological roles, including transporting nutrients through TonB-dependent transporters (TBDTs) and maintaining membrane integrity during cell division (49, 50). Like other group B colicins, namely, ColD (H^17^SMVV^21^) (51), ColM (E^2^TLTVH^7^) (52, 53), and klebicin C (M^16^VSLG^20^) (17), KlebE also contains a TonB-box (E^3^ETLTVV^9^) in its disordered region that is essential for its cytotoxicity. The dissimilar phenotypes resulting from KlebE exposure between the double-gene mutant (Δ*exbB*Δ*exbD*) and the quadruple mutant (Δ*exbB*Δ*exbD*Δ*tolQ*Δ*tolR*) suggested that *tolQ/R* could replace *exbB/D*, albeit at a low level. Cross-complementation between the two gene pairs was also found for other group B colicins (ColB and Col5) and group A colicins; nevertheless, this is not possible with TolA and TonB (54, 55).

Parasitizing bacterial OMPs is necessary for toxin recognition by the Ton or Tol system. A receptor and translocator model has recently been demonstrated for toxin transfer from the OM to the periplasm (56). Group A toxins typically utilize their N-terminal translocation domain to bind to a porin, often OmpF, or the drug efflux channel TolC. This process allows the threading of disordered epitopes across the porin channel after a TBDT or the porin itself is recognized through the receptor domain for initial attachment. For example, in the case of E group colicins (ColE2-E9) (30, 57), BtuB serves as the receptor, and OmpF serves as the translocator; in the case of ColN (35), OmpF serves as both the receptor and translocator; and in the case of ColE1 (58), BtuB serves as the receptor, while TolC serves as the translocator. Group B toxins are usually transported in an unfolded state through TBDTs after docking on the bacterial surface. Typically, the pyocins S2 and S5 both associate with common polysaccharide antigens as the primary receptor and then exploit the pyoverdine transporter FpvAI and the ferric pyochelin transporter FptA for translocation, respectively (30, 48). An exception to the group B bacteriocins that were recently uncovered is TolC, which acts as both a receptor and translocator for klebicin C import (17). Our studies suggest that the susceptibility of Ton-dependent KlebE is dependent on the porin OmpC rather than OmpF. Although both OmpC and OmpF are abundant trimeric β-barrel OMPs with narrow channels, there are few reports on the utilization of OmpC by colicin or colicin-like bacteriocins compared to OmpF (18). Furthermore, the involvement of porins is a fundamental aspect of the import mechanism of Group A bacteriocins (35). Our research suggested that OmpC is another crucial receptor for bacteriocin import, indicating that porins are not solely utilized by Group A toxins.

The specific receptors and Ton or Tol proteins utilized by colicins are responsible for the specificity of their activity spectra. The *E. coli* strain was engineered to be susceptible to pyocin S5 by expressing FptA and TonB1 from *P. aeruginosa* (48). Three regions of OmpF (residues 1–63, 115–262, and 279–297) are involved in colicin N entry (59). Even one amino acid mutation (Gly119) in internal loop 3 of OmpF was found to confer colicin N-resistance due to lumen constriction (60). Our study also revealed that the single 91st aspartic acid of OmpC plays a decisive role in the toxin killing effect; moreover, this residue resides in the external loop. We predicted that this residue mutation would block the initial binding of the KlebE to the outer membrane of the target bacteria. It was recently found that the extracellular PDZ domain of the receptor RseP is the key determinant of both binding and killing by the bacteriocin EntK1 of *Enterococcus faecium* (61). Additionally, complementation of OmpC from *K. pneumoniae* provides *E. coli* susceptibility to KlebE, suggesting that OmpC is likely both the receptor and translocator, but this needs to be further elucidated. Obviously, a few residue mutations in the receptor are enough to change the sensitivity of bacteriocins. Unfortunately, porins undergo rapid mutational alterations, especially in the external loops between transmembrane strands, as they interact with elements of the external world, such as antibodies, components of the innate immune system, bacteriocins, and phages (62); thus, the use of bacteriocins in rational combinations (cocktails) should be exploited to cure multidrug-resistant infections. Uncovering more bacteriocins from natural isolates can undoubtedly favor the design of cocktails.

In conclusion, this study identified KlebE, which is a small bacteriocin produced by *K. pneumoniae*. This molecule mediates interbacterial competition and has a broad and potent bactericidal effect on MDR *K. pneumoniae* isolates. KlebE, which comprises three typical domains—a disordered N-terminal domain, a globular central domain, and a colicin A-like toxic domain—significantly disrupts the integrity of the bacterial cell membrane and causes transmembrane defects in artificial lipid membranes, validating its classification as a pore-forming toxin. Bacteriocins have evolved to exploit OMPs for their import and antibacterial activity. The toxicity of KlebE was shown to rely on Ton proteins and the porin protein OmpC. The TonB-box, a 7-residue sequence (E^3^ETLTVV^9^) located within the disordered region, is responsible for the interaction between KlebE and TonB, with L6 and V8 being the most essential amino acids. Additionally, residues 150 to 159 in KlebE are required for the interaction between KlebE and OmpC. Eight residues, namely, K92, F93, K131, E132, G152, G153, H159, and M161, which are predominantly located in two disordered regions of the central domain, were found to be essential for KlebE toxicity; notably, K92 and G152 exhibited the most pronounced impact. Finally, the 91st residue of OmpC, an aspartic acid residue, was found to be key for KlebE toxicity. This study confirmed a bacteriocin in *K. pneumoniae* and elucidated its mode of action, associated cell membrane proteins and interaction domains, providing a foundation for the development of bacteriocin-based alternative antibiotic therapies.

## Experimental procedures

### Bacterial strains and culture conditions

The bacterial strains and plasmids used in this study are described in Tables S1–S3. *K. pneumoniae* and *E. coli* were cultured in LB broth or agar. Bacteria were cultured at 37 °C and 180 rpm. When necessary, antibiotics and diaminopimelic acid (DAP) were added to the medium at the following concentrations: kanamycin at 50 μg/ml, ampicillin at 100 μg/ml, chloramphenicol at 25 μg/ml, spectinomycin at 80 μg/ml, tetracycline at 50 μg/ml, and DAP at 50 μg/ml. LB agar containing 12.5% sucrose was used for *sacB* gene-based counterselection in the allelic exchange experiments.

### Bacterial isolation

Respiratory tract samples were collected from duck or goose farms in Sichuan Province, China. Each specimen was separated using the following standard procedure with minor modifications (63). In brief, samples were added to 10 ml of buffered peptone preenrichment solution and incubated at 37 °C for 24 h. Then, 100 μl of culture medium was streaked onto blood agar overnight. Samples were considered culture-positive if one or more colonies were observed. Based on the colony morphology, one colony from each sample was subcultured by streaking on blood agar and MacConkey plates. An additional subculture was conducted if different morphological colonies grew on a plate. Putative *K. pneumoniae* colonies were selected and subjected to polymerase chain reaction (PCR) identification using the 16S primers 16S-F/R (16S-F: AGAGTTTGATCCTGGCTCAG, 16S-R: AGAGTTTGATCCTGGCTCAG). Amplicons were sequenced at the Genomic Sequencing Laboratory (Sangon Biotech, Shanghai, China). All identified isolates were stored in 15% (v/v) glycerol at −80 °C.

### Genome database mining to identify putative bacteriocins

The complete genomes of 61 *Salmonella* strains were sequenced in our previous study (21). To obtain genetic information for 7 *K. pneumoniae* isolates, we extracted high-quality genomic and plasmid DNA using a TIANamp Bacteria DNA Kit (Tiangen Biotech, Beijing, China). Genomic sequencing of 7 *K. pneumoniae* isolates was conducted using an MGISEQ-2000 system (MGI Tech, Shenzhen, China). SPAdes (64) v3.15.3 was used to assemble the filtered subreads. Gene prediction was performed using Prokka (65) v1.11. Annotation was performed using BLAST with the eggNOG database (66). Genes potentially related to bacteriocin were identified using BACTIBASE (22) (http://bactibase.hammamilab.org/), Bagel4 (23) (http://bagel4.molgenrug.nl/), and APD3 (24) (https://wangapd3.com/main.php).

For identification of candidate bacteriocin genes, the gene sequences (either individual candidate toxins or bacteriocin-immunity pairs) were amplified and inserted into pET-28a vectors (BBI, Shanghai, China) at the *Nco*I and *Xho*I sites with the SeamLess Cloning ligase (BBI). Subsequently, the recombinant plasmid or the control pET-28a plasmid was transformed into the *E. coli* BL21 (DE3) strain. Overnight cultures of the *E. coli* strains were diluted to an optical density of 600 nm (OD_600_) = 0.1 in LB medium. Thereafter, all the cultures were incubated at 37 °C with shaking at 180 rpm for 3 h in the presence of 0.1 mM IPTG. The cultures were then serially diluted 10-fold and spotted on LB agar plates. Subsequently, all plates were incubated overnight at 37 °C to observe bacterial growth.

### The MICs of antibiotics

The MICs of 10 antibiotics for 19 *K. pneumoniae* isolates were determined by the broth dilution method (67). The antimicrobial agents employed were as follows: ampicillin, cefoxitin, cefotaxime, cefepime, aztreonam, azithromycin, kanamycin, ofloxacin, tetracycline, and polymyxin. The *E. coli* reference strain ATCC 25922 was used for quality control. Susceptibility, intermediate resistance or resistance were defined according to the Clinical and Laboratory Standards Institute (CLSI) guidelines (68). Multidrug resistance was defined as acquired nonsusceptibility to at least one agent in three or more antimicrobial categories (69).

### Protein expression and purification

The primers used in this study are listed in Table S4. For expression of KlebE_-His_, the gene was amplified with the primers pET28a-KlebE-F/R. The PCR product was inserted into the pET-28a vector (BBI) between the *Nco*I and *Xho*I sites with SeamLess Cloning ligase (BBI), generating the recombinant plasmid pET28a-KlebE. The resulting plasmid was subsequently transformed into *E. coli* BL21 (DE3) for protein expression. The same method was used to construct *E. coli* strains expressing truncated KlebE_-His_, point mutants of KlebE_-His_, and OmpC_-Flag_. Additionally, the plasmid pBAD24 was used as a carrier for OmpC_Kp003_, OmpC_Kp002_, or OmpC_Kp002_, with multipoint or single-point mutations, in BL21 (DE3) cells; the plasmid pCold (TaKaRa, Tokyo, Japan) was used for expressing TonB_-Flag_. The tags, including His and Flag, were added at the C-terminus of the target proteins.

Recombinant *E. coli* BL21 (DE3) cells were cultured in LB medium supplemented with kanamycin or ampicillin. Protein expression was induced during the logarithmic phase (OD_600_ of 0.8) with 1.0 mM IPTG for 4 h. The cells were harvested by centrifugation at 5000 rpm for 10 min. Afterward, the cells were washed twice with 10 mM PBS and resuspended in lysis buffer containing imidazole (10 mM imidazole, 50 mM NaH_2_PO_4_, 300 mM NaCl [pH 8]). The pellets were sonicated on ice using an ultrasonic cell disruptor (XianChang, Ningbo, China) at a frequency of 30 kHz for 20 min (5 s off, 5 s on), and the supernatant was collected after centrifuging the pellets at 12,000 rpm for 10 min at −4 °C. Ni-NTA affinity purification was used to purify the proteins. Before loading, a Ni-NTA Beads 6FF Gravity column (Smart Lifescience, Changzhou, China) was equilibrated with 5 column volumes of cold lysis buffer. The proteins were fully bound to Ni-Sepharose, after which the column was washed with wash buffer (20 mM imidazole, 50 mM NaH_2_PO_4_, 300 mM NaCl [pH 8]). Subsequently, the proteins were eluted using the same buffer containing 250 mM imidazole. The proteins containing His_6_-tags were collected and resuspended in PBS using 10 kDa AMICON Ultra15 Centrifugal Filter Units (Millipore, Massachusetts, America) at 4500*g* for 30 min at 4 °C. The purity of the proteins was assessed using SDS‒PAGE, and the concentrations of the proteins were determined using a bicinchoninic acid protein assay kit (Thermo Fisher Scientific, Massachusetts, America). All proteins were filter sterilized through 0.22-mm-pore filter units (Millipore) and stored in 20% (v/v) glycerol at 4 °C for subsequent experiments.

### Antibacterial activity of KlebE or modified KlebE

*K. pneumoniae* isolates or *E. coli* BL21 (DE3) cells were grown to OD_600_ = 0.8 in LB media and diluted 100-fold in 0.75% top agar preheated in a 55 °C water bath. The mixed overlay components were poured onto plates containing solid medium (LB containing 1.5% [w/v] agar) in round or square Petri dishes. To determine the antimicrobial efficacy of KlebE, sterile Whatman discs (6 mm diameter) were placed on soft agar, and 20 μl of purified KlebE (40 μg) was added to the disks. The negative control consisted of a BSA standard solution (2 mg/ml, BBI) with the Kp002 strain as the indicator. For other assays of antibacterial activity using the soft agar overlay method, slight modifications were made where 10 μl of KlebE or modified KlebE at different concentrations was precisely spotted onto plates with the indicator Kp002 or its mutant strains or with BL21 (DE3) cells or its derivatives expressing OmpC from *K. pneumoniae*. The plates were incubated overnight at 37 °C, after which the inhibition zone diameters were measured.

### Construction of the *K. pneumoniae* mutant and complemented strains

*K. pneumoniae* mutant strains were constructed by allelic exchange using the suicide vector pRE112 as previously described (70). For deletion of the gene *Kp006_4991* in Kp006, the primer pairs D*Kp006_4991*-F1/R1 and D*Kp006_4991*-F2/R2 were used to amplify the regions ∼400 bp upstream and downstream of the target gene, respectively. The two fragments were subsequently joined by PCR using the primers D*Kp006_4991*-F1/R2. The resulting PCR product was digested and ligated into the plasmid pRE112 with Seamless Cloning ligase (BBI) to generate the plasmid pRE112-Δ*Kp006_4991*, which carries a deletion of the entire *Kp006_4991* gene sequence. This plasmid was subsequently introduced into Kp006 by *E. coli* SM10 λ *pir* (71) *via* conjugation. Recipient cells were subjected to chloramphenicol-based positive screening and sucrose-based counterselection to generate marker-free gene mutations. The *Kp006_4991* mutant was ultimately confirmed by PCR using the primers D*Kp006_4991*-F1/R2. For gene complementation, the coding sequence of *Kp006_4991* was amplified using the C*Kp006_4991*-F/R primer pair and subsequently cloned and inserted into the pET28a plasmid to generate the complementation plasmid C-*Kp006_4991*-pET28a. Finally, the plasmid was transformed into the corresponding mutant strain, generating the complemented strain Δ*4991*-Kp006 (*4991*). The same method was used to delete other genes and construct complemented strains.

### Intraspecies competition assay of *K. pneumoniae*

Interbacterial competition was detected in a coculture system as previously described (72). Two target competing bacterial strains, the producer and the indicator, were statically grown. The overnight culture was diluted 1:100 in fresh LB medium, and growth was continued at 37 °C and 180 rpm until an OD_600_ of 0.8 was reached. Then, equal volumes of the two bacterial cultures were added to 5 ml of LB liquid medium at a ratio of 1:1000 and incubated together for 12 h at 180 rpm and 37 °C. Then, 0.5 mM IPTG was added to the medium to induce gene expression in experiments involving complementation plasmids. Finally, the CFUs of the competing strains were quantified on solid LB plates containing the appropriate antibiotics (spectinomycin, tetracycline, or kanamycin) using droplet plate counting at time points 0 h and 12 h. The competitive index was calculated as the final frequency of the indicator divided by its initial frequency in the mixed cocultures.

CI = lg CFU [(indicator_T12_/producer_T12_)/(indicator_T0_/producer_T0_)]

### Evaluation of the antibacterial performance of KlebE

For measurement of the toxin-killing curve, overnight cultures of the indicator Kp002 were subcultured to OD_600_ = 0.8 in LB medium. Then, the LB medium was supplemented with different doses of the KlebE protein (0, 0.125, 0.25, 0.5, 1, 2, 4, 8, 16, 32, or 64 μg/ml) to determine the correlation between concentration and bactericidal bioactivity. Thereafter, all the mixtures were incubated without shaking at 37 °C for 1 h. The samples were then serially diluted 10-fold and applied onto LB agar plates. The plates were incubated overnight at 37 °C to determine the CFU. To determine the relationship between time and bactericidal bioactivity, the indicator strain was incubated on a shaker until the OD_600_ reached 0.1. Then, 16 μg/ml of KlebE or an equal volume of buffer without proteins was incubated with the bacterial culture mixture at 37°C with shaking at 180 rpm. Afterward, the OD_600_ was measured every hour for a period of 8 h using a UV spectrophotometer.

### N01 and PI staining assay

The Kp002 indicator in the logarithmic phase was incubated with 16 μg/ml KlebE or protein-free sterile PBS for 1 h at 37 °C. Then, for measurement of the membrane permeability (73), cells were stained with the probes N01 and PI by using the BBcell Probe live/dead bacterial staining kit (BestBio). Briefly, 1 ml of the bacterial mixture was washed and resuspended in 0.85% NaCl, followed by the addition of a 12 μl mixture of N01 and PI (1:5). The samples were incubated in the dark at 24 °C for 15 min and then visualized using an inverted fluorescence microscope system (Olympus BX53).

### SEM and TEM observations

Kp002 cells were treated with KlebE according to the N01 and PI staining assay protocols. The cells were then fixed with 2.5% glutaraldehyde. For SEM observation, cells were immobilized on a glass slide coated with poly-L-lysine. Secondary fixation was conducted in a mixture of 1.5% potassium ferricyanide and 1.0% osmium tetroxide. Prior to dehydration with hexamethyldisilane, the samples were predehydrated using ethanol at 50%, 60%, 70%, 80%, 90%, and 95% in series. The dehydrated samples were sputter coated with gold at a 30 mA plasma current under argon gas for 150 s (Hitachi MC1000). Microscopic examinations were carried out using SEM (Hitachi SU8100) at an acceleration voltage of 10 kV. For TEM observation, the cells were washed with anhydrous ethanol several times at 24 °C for dehydration. The samples were embedded, polymerized, and subsequently sliced into 70- to 100-nm-thick sections using an ultramicrotome (Leica). Thereafter, the samples were deposited on carbon-coated copper grids. Finally, the morphology of the bacterial cells was observed *via* TEM (Hitachi H-7650).

### Dynamic GUV leakage assay

The GUV leakage assay was conducted as previously described (74). In brief, GUVs were prepared following the conventional electroformation method (75). A mixture of PC and PG lipids (PC/PG = 4/1 by mol) labeled with 0.1 wt% rhodamine B-phosphoethanolamine was predissolved in chloroform at a concentration of 2.0 mg/ml. 20 microliters of the solution was deposited onto indium tin oxide-coated glass slides and dried under vacuum overnight. The dry film was transferred to a custom electroformation chamber (with the two glass slides serving as electrodes) and rehydrated in 0.1 M sucrose buffer. Alternating voltages were applied (0.5 V × 20 min, 1.0 V × 20 min, and 1.5 V × 3 h). The obtained vesicles were washed three times *via* centrifugation (8000 rpm for 20 min). Well-dispersed GUVs with a size distribution of 8 to 30 μm were collected and redispersed in 0.1 M glucose buffer containing 0.2 mg/ml calcein (with a final lipid concentration of ≈ 0.02 mg/ml).

The prepared GUV and calcein mixture was transferred to a custom-made chamber cell with a cover-glass substrate for microscopic observation. For vesicle immobilization, the glass substrate was washed carefully, dipped into aminopropyltriethoxysilane for 5 min, and dried under N_2_ flow. Afterward, the glass slide was held at 120 °C for 30 min before being placed in the chamber for use. For *in situ* microscopy observations, a volume of the GUV dispersion was transferred to the chamber cell and stabilized for approximately 5 min for particle immobilization.

After the immobilization of the GUVs on the substrate, KlebE solution was added at concentrations of 0, 64, or 160 μg/ml, and the dynamic entry of calcein into the interior of the GUV at each time point was subsequently observed by an inverted confocal laser scanning microscope (Nikon AX R) equipped with a 63× oil objective. Signals from the Lissamine rhodamine B channel for lipids (EX 561 nm, EM 593/46 nm), the calcein channel (EX 488 nm, EM 525/50 nm), and the overlaid channel were simultaneously captured. All images were captured under the same instrumental settings. The fluorescence intensity of the GUV interior at each time point (mean value among pixels read directly from the software) was normalized to that of the surrounding environment and plotted as a function of time. Three parallel experiments were performed for each KlebE treatment, and representative GUVs are shown in the main text.

### Pulldown assay and Western blotting analysis

The interactions between KlebE or mutant KlebE and target proteins were evaluated using pulldown assays and Western blot analysis. In brief, *E. coli* BL21 (DE3) cells harboring either the empty plasmid or recombinant plasmids (TonB-pCold or OmpC-pET28a) were cultured until they reached the logarithmic phase, followed by incubation with 0.5 mM IPTG for 12 h at 16 °C. Subsequently, the *E. coli* cells were harvested and lysed using lysis buffer (150 mM NaCl, 20 mM Na_2_HPO_4_ [pH 7]). Afterward, the lysate was mixed with 10 μg of rabbit anti-His_6_-tag mAb (10001-0-AP; Proteintech, Illinois, America) and 60 μg of purified KlebE or mutant KlebE in a total volume of 1.0 ml and incubated at 4 °C for 12 to 24 h. After incubation, 100 μl of Protein A/G magnetic beads (Smart Life Science) was added to perform a pulldown experiment at 24 °C for 2 h. Subsequently, equal amounts of bead samples were separated *via* SDS‒PAGE after washing with precooled wash buffer and analyzed *via* Western blotting with a mouse anti-His_6_-tag mAb (66005-1-Ig, Proteintech) and a mouse anti-Flag-tag mAb (M185-3LL, MBL). The specific KlebE bands produced by the anti-His_6_ mAb were consistent with the amounts of the group samples loaded. Additionally, for identification of KlebE-associated membrane proteins, 200 μg of purified KlebE_-His_ was incubated with lysed whole-cell proteins from the indicator strain Kp002, followed by protein A/G affinity chromatography, SDS‒PAGE analysis, and peptide mass fingerprinting.

### Point mutations of KlebE or OmpC

For point mutations in the N-terminal region of KlebE or OmpC, the fragment containing the point mutation was amplified by changing the target amino acid to GCA (alanine codon) or GGA (glycine codon) on the forward primers. To construct point mutants in the central domain of KlebE or OmpC, we utilized a three-step PCR approach. For the G91A mutation in KlebE, the primer pairs pET28a-KlebE-F/G91A-KlebE-R1 and G91A-KlebE-F2/pET28a-KlebE-R were used to amplify the upper and lower segments of the KlebE gene, respectively. The target site amino acids for the R1 and F2 primers were designed to be either GCA or GGA. Subsequently, these two fragments were joined together by PCR using the primers pET28a-KlebE-F/R followed by ligation with SeamLess Cloning ligase (BBI) to generate the plasmid G91A-KlebE-pET28a for protein expression. The same method was used to construct other point mutations in KlebE or OmpC.

### Structure and statistical analysis

The tertiary structures of KlebE and ColA were predicted using AlphaFold2 (29). The transmembrane region of the immunity protein Kp006_4990 was predicted *via* TMpred. Additionally, we used ProteinsPlus (38) to predict and analyze the potential binding pockets of KlebE. All model drawings were made using PyMOL (DeLano Scientific LLC). The data are shown as the means ± SD of three independent biological replicates and were analyzed by two-tailed Student’s *t* test or one-way ANOVA followed by Dunnett's multiple comparison test in GraphPad Prism (GraphPad Software). A probability value of *p* < 0.05 was considered to indicate statistical significance. The *in vitro* experiments were independently conducted three times.

## Data availability

All the data are contained within the manuscript. The genome or gene sequences can be found in the NCBI database with the indicated accession numbers within the manuscript.

## Supporting information

This article contains supporting information.

## Conflict of interest

The authors declare that they have no conflicts of interest regarding the contents of this article.
