# Supplementary material for: Klebicin E, a pore-forming bacteriocin of Klebsiella pneumoniae, exploits the porin OmpC and the Ton system for translocation
Source: J Biol Chem. 2024 Jan 30;300(3):105694. doi: 10.1016/j.jbc.2024.105694 (PMC10906532; doi:10.1016/j.jbc.2024.105694)
Supplement: Supporting Information [file mmc1.docx]

**Klebicin E, a pore-forming bacteriocin of *Klebsiella pneumoniae*, exploits the porin OmpC and Ton system for translocation**

Xinxin Zhao^1,2,3,4^, Wenyu Wang^3,#^, Xiaoli Zeng^3^, Rong Xu^5^, Bing Yuan^5^, Wenyao Yu^3^, Mingshu Wang^1,2,3,4^, Renyong Jia^1,2,3,4^, Shun Chen^1,2,3,4^, Dekang Zhu^1,2,3,4^, Mafeng Liu^1,2,3,4^, Qiao Yang^1,2,3,4^, Ying Wu^1,2,3,4^, Shaqiu Zhang ^1,2,3,4^, Juan Huang ^1,2,3,4^, Xumin Ou ^1,2,3,4^, Di Sun^1,2,3,4^, Anchun Cheng ^1,2,3,4*^

^1^ Research Center of Avian Diseases, College of Veterinary Medicine, Sichuan Agricultural University, Chengdu, Sichuan, China

^2^ Key Laboratory of Animal Disease and Human Health of Sichuan Province, Chengdu, Sichuan, China

^3^ Institute of Veterinary Medicine and Immunology, College of Veterinary Medicine, Sichuan Agricultural University, Chengdu, Sichuan, China

^4^ Engineering Research Center of Southwest Animal Disease Prevention and Control Technology, Ministry of Education of the People's Republic of China, Chengdu, Sichuan, China

^5^ Songshan Lake Materials Laboratory, Dongguan 523808, Guangdong, China

^*^ Correspondence: Anchun Cheng, chenganchun@vip.163.com.

^#^ These authors have contributed equally to this work.

**Supporting Information**

**Supplement file 1 contains:**

Table S1. *K. pneumoniae* strains isolated from ducks or geese

Table S2. Bacterial strains in this study

Figure S1. Sequence alignments of bacteriocins

Figure S2. Sequence alignments of immunity proteins.

Figure S3. The MIC of antibiotics to *K. pneumoniae* isolates

Figure S4. SDS-PAGE analysis of purified KlebE

Figure S5. Protein pocket P_10 pattern predicted by ProteinsPlus

Figure S6. Sequence alignments of *K. pneumoniae* OmpC

References

**Supplement file 2**

Table S3. Plasmids used in this study

**Supplement file 3**

Table S4. Primers used in this study

**Table S1. *K. pneumoniae* strains isolated from ducks or geese**

| **Strain name** | **Species** | **Source** | **Region** | **Year** | **Genomes** |
| --- | --- | --- | --- | --- | --- |
| Kp001 | *K. pneumoniae* | duckling respiratory tract | Sichuan, China | 2019 | JAUPSV000000000 |
| Kp002 | *K. pneumoniae* | duckling respiratory tract | Sichuan, China | 2019 | JAUPTB000000000 |
| Kp003 | *K. pneumoniae* | duckling respiratory tract | Sichuan, China | 2019 | JAUPTA000000000 |
| Kp004 | *K. pneumoniae* | duckling respiratory tract | Sichuan, China | 2019 | JAUPSZ000000000 |
| Kp005 | *K. pneumoniae* | duckling respiratory tract | Sichuan, China | 2019 | JAUPSY000000000 |
| Kp006 | *K. pneumoniae* | duckling respiratory tract | Sichuan, China | 2019 | JAUPSX000000000 |
| Kp007 | *K. pneumoniae* | duckling respiratory tract | Sichuan, China | 2019 | JAUPSW000000000 |
| Kp008 | *K. pneumoniae* | goose respiratory tract | Sichuan, China | 2022 | ND |
| Kp009 | *K. pneumoniae* | goose respiratory tract | Sichuan, China | 2022 | ND |
| Kp010 | *K. pneumoniae* | goose respiratory tract | Sichuan, China | 2022 | ND |
| Kp011 | *K. pneumoniae* | goose respiratory tract | Sichuan, China | 2022 | ND |
| Kp012 | *K. pneumoniae* | duck respiratory tract | Sichuan, China | 2023 | ND |
| Kp013 | *K. pneumoniae* | duck respiratory tract | Sichuan, China | 2023 | ND |
| Kp014 | *K. pneumoniae* | duck respiratory tract | Sichuan, China | 2023 | ND |
| Kp015 | *K. pneumoniae* | duck respiratory tract | Sichuan, China | 2023 | ND |
| Kp016 | *K. pneumoniae* | duck respiratory tract | Sichuan, China | 2023 | ND |
| Kp017 | *K. pneumoniae* | duckling respiratory tract | Sichuan, China | 2023 | ND |
| Kp018 | *K. pneumoniae* | duckling respiratory tract | Sichuan, China | 2023 | ND |
| Kp019 | *K. pneumoniae* | duckling respiratory tract | Sichuan, China | 2023 | ND |

ND, not detected.

**Table S2. Bacterial strains in this study**

| **Strain** | **Description** | **Source** |
| --- | --- | --- |
| BL21(DE3) | *E. coli*; Expressing host cell | Sangon Biotech |
| SM10 λ pir | *E. coli*; Suicide plasmid cloning host cell | (1) |
| Δ*4991*-Kp006 | Kp006 *Kp006_4991(KlebE)* gene mutant | this study |
| Δ*ompA*-Kp002 | Kp002 *ompA* mutant strain | this study |
| Δ*ompC*-Kp002 | Kp002 *ompC* mutant strain | this study |
| Δ*ompF*-Kp002 | Kp002 *ompF* mutant strain | this study |
| Δ*ompX*-Kp002 | Kp002 *ompX* mutant strain | this study |
| Δ*fhuA*-Kp002 | Kp002 *fhuA* mutant strain | this study |
| Δ*fiu*-Kp002 | Kp002 *fiu* mutant strain | this study |
| Δ*fcuA*-Kp002 | Kp002 *fcuA* mutant strain | this study |
| Δ*tolC*-Kp002 | Kp002 *tolC* mutant strain | this study |
| Δ*Tol-Pal*-Kp002 | Kp002 *tolA/B/Q/R* and *pal* mutant strain | this study |
| Δ*btuB*-Kp002 | Kp002 *btuB* mutant strain | this study |
| Δ*tonB*-Kp002 | Kp002 *tonB* mutant strain | this study |
| Δ*exbB*-Kp002 | Kp002 *exbB* mutant strain | this study |
| Δ*exbD*-Kp002 | Kp002 *exbD* mutant strain | this study |
| Δ*exbB*Δ*exbD*-Kp002 | Kp002 *exbB* and *exbD* mutant strain | this study |
| Δ*exbB*Δ*exbD*Δ*tolQ*Δ*tolR*-Kp002 | Kp002 *exbB, exbD, tolQ, tolR* mutant strain | this study |
| Kp002 (*4990*) | Kp002 carrying pET28a-Kp006_4990 | this study |
| Δ*4991*-Kp006 (*4991*) | Δ*4991*-Kp006 carrying pET28a-Kp006_4991 | this study |

**
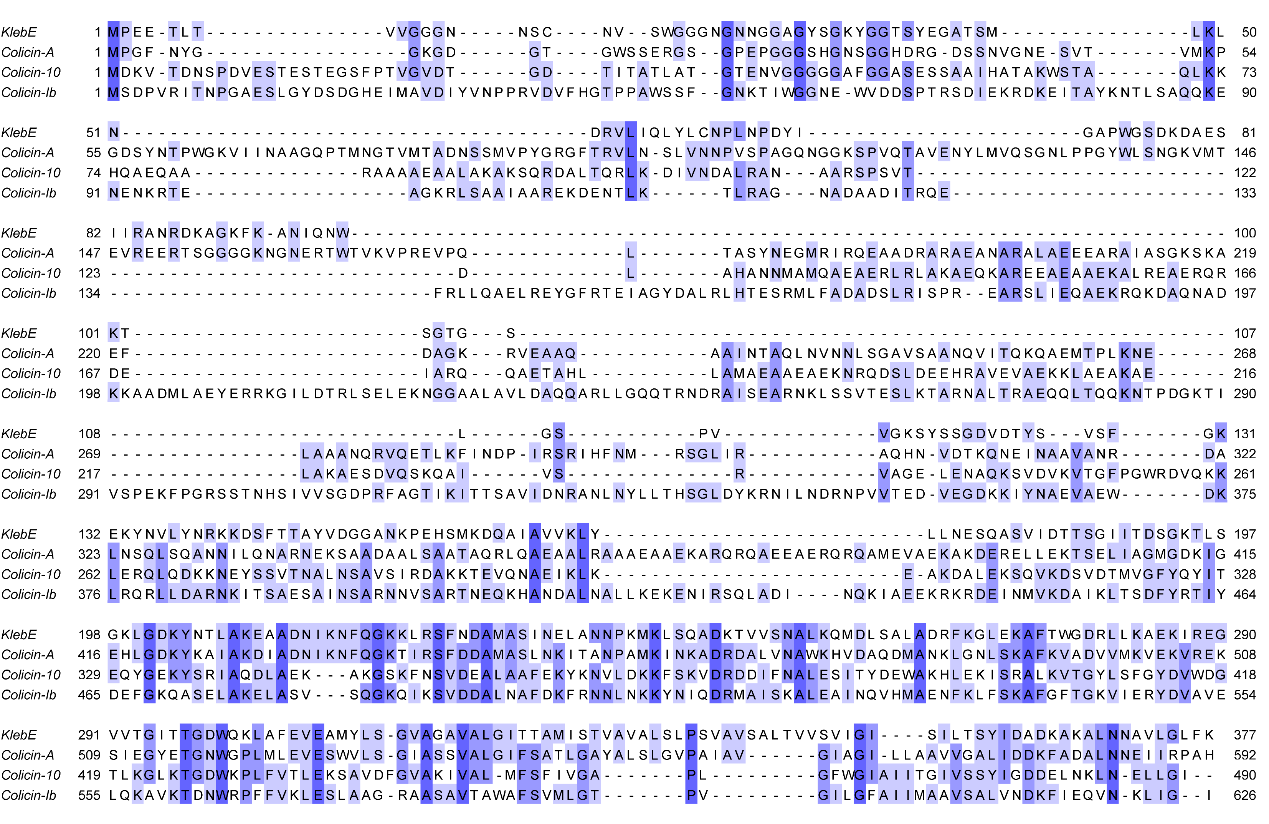
Figure S1.** **Sequence alignments of bacteriocins.** Amino acid sequence alignments of Kp006_4991 (GenBank: MEC7341763.1) with ColA (Uniport: P04480), Col10 (Uniport: Q47125) and ColⅠb (Uniport: P04479). The resemblance (blue) between these sequences was predominantly concentrated within the C-terminal toxic domain.

**
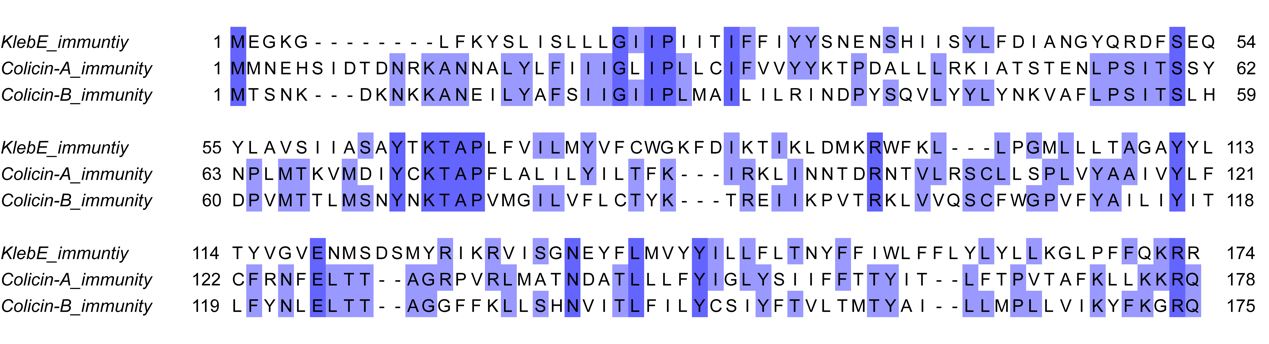
Figure S2. Sequence alignments of immunity proteins.** Amino acid sequence alignments of Kp006_4990 (GenBank: MEC7341762.1) with the ColA immunity protein (Uniport: P05701) and ColB immunity protein (Uniport: P22426).


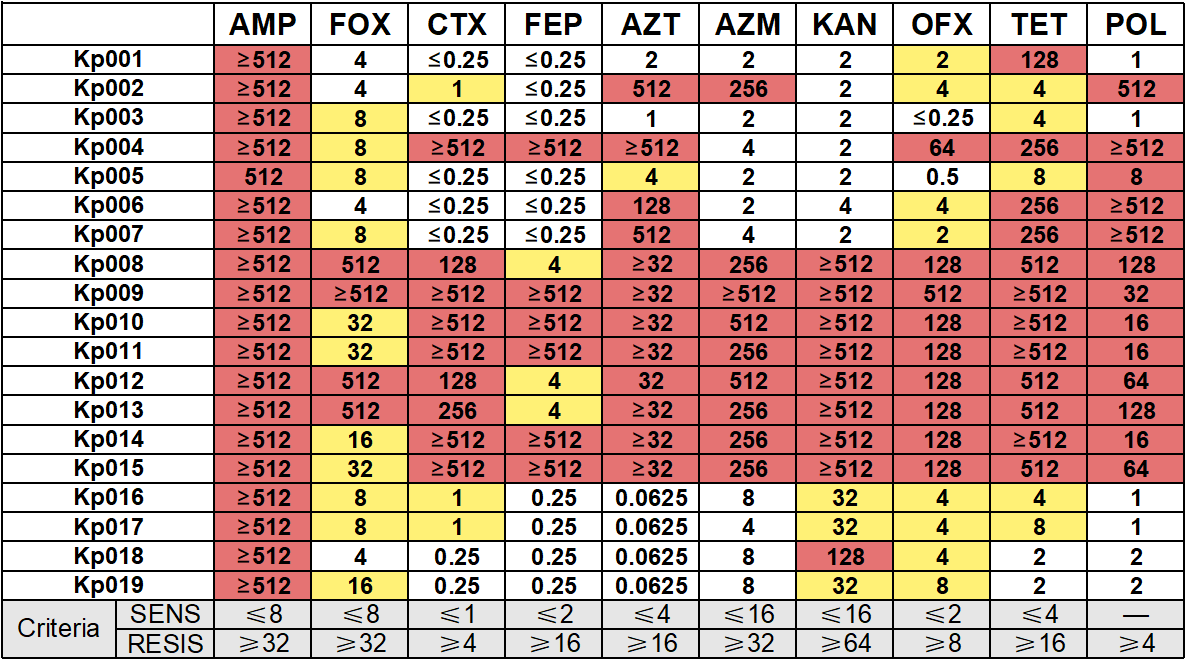


**Figure S3.** **The MIC of antibiotics to *K. pneumoniae* isolates.** The MIC was defined as the lowest antibiotic concentration at which there was no visible growth of bacteria after 12 hours incubation. The white indicates sensitivity; yellow indicates intermediate resistance; red indicates resistance. AMP, Ampicillin; FOX, cefoxitin; CTX, cefotaxime; FEP, cefepime; AZT, aztreonam; AZM, azithromycin; KAN, kanamycin; OFX, ofloxacin; TET, tetracycline; POL, polymyxin. SENS, sensitivity; RESIS, resistance.


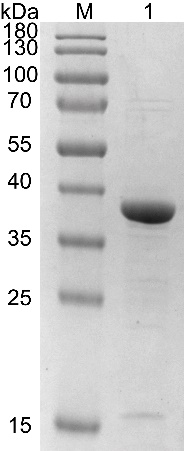


**Figure S4. SDS-PAGE analysis of purified KlebE.** Lanes: M, 180-kDa prestained protein marker; 1, purified KlebE.

**
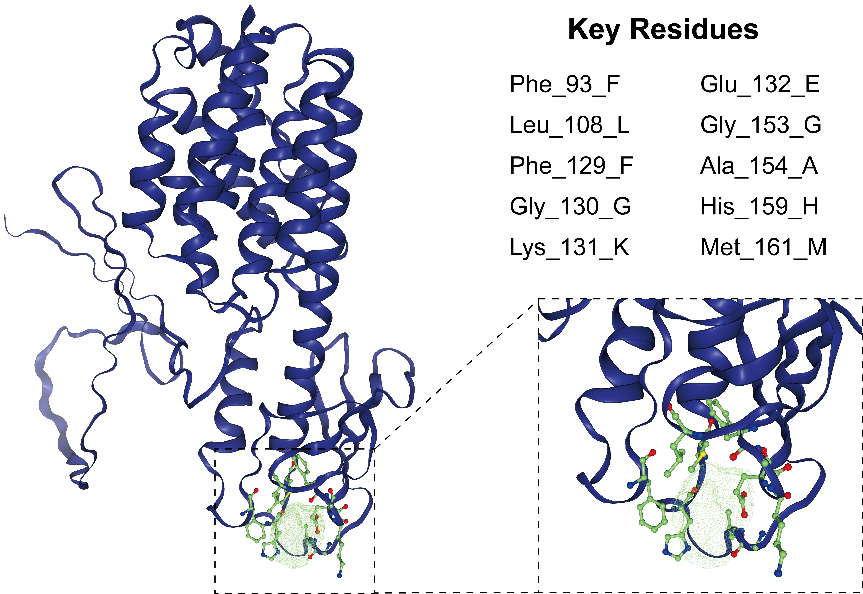
**

**Figure S5.** **Protein pocket P_10 pattern predicted by ProteinsPlus.** Size and shape descriptors: volume [Å³], 166.78; surface [Å²], 316.30; depth [Å], 8.66. P_10 protein pockets contain key amino acid sites: F93, L108, F129, G130, K131, E132, G153, A154, H159, and M161.

**
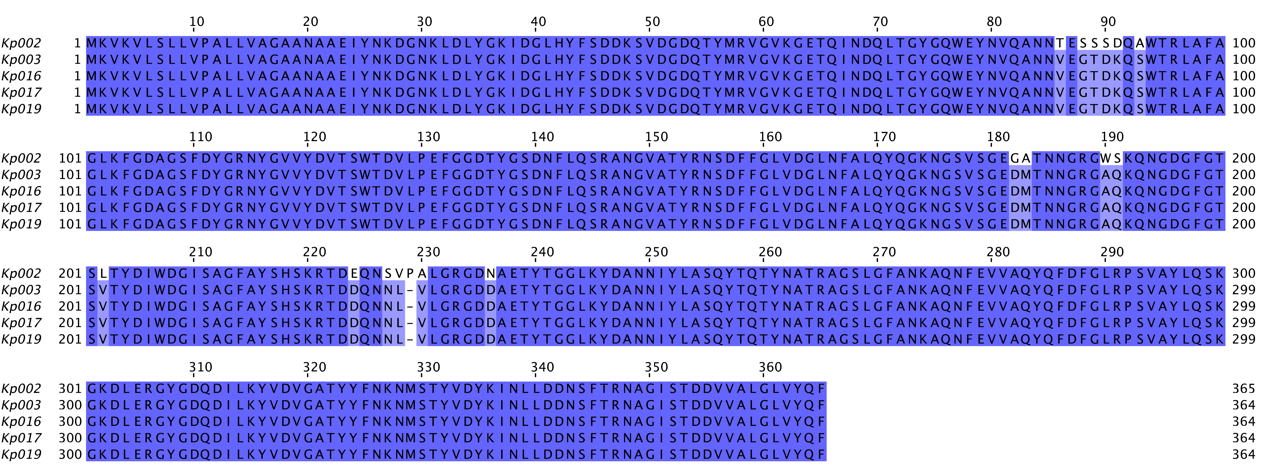
**

**Figure S6. Sequence alignments of *K. pneumoniae* OmpC.** Amino acid sequence alignments of OmpC between the indicator strain Kp002 (GenBank: MEC7343648.1) and four KlebE-insusceptible strains, namely, Kp003, Kp016, Kp017 and Kp019 (GenBank: MEC7310612.1).

**References**

1. Rubirés, X., Saigi, F., Piqué, N., Climent, N., Merino, S., Albertí, S. *et al.* (1997) A gene (wbbL) from Serratia marcescens N28b (O4) complements the rfb-50 mutation of Escherichia coli K-12 derivatives J Bacteriol **179**, 7581-7586 10.1128/jb.179.23.7581-7586.1997

(2)
